# Thermally Drawn Multifunctional All‐Hydrogel Fibers for Anti‐Fibrotic and Multimodal Neural Interfaces

**DOI:** 10.1002/adma.202511634

**Published:** 2025-10-09

**Authors:** Changhoon Sung, Kum Seok Nam, Yeji Kim, Honey Kang, Kanghyeon Kim, Chanwoong Yoon, Somin Lee, Ain Chung, Jiheong Kang, Young‐Gyun Park, Alan Jung Park, Haider Butt, Hyunwoo Yuk, Seongjun Park

**Affiliations:** ^1^ Medical Research Center Seoul National University Seoul 03080 Republic of Korea; ^2^ Department of Bio and Brain Engineering Korea Advanced Institute of Science and Technology (KAIST) Daejeon 34141 Republic of Korea; ^3^ Department of Biomedical Sciences College of Medicine Seoul National University Seoul 03080 Republic of Korea; ^4^ Program of Brain and Cognitive Engineering Korea Advanced Institute of Science and Technology (KAIST) Daejeon 34141 Republic of Korea; ^5^ Department of Chemistry Seoul National University Seoul 03080 Republic of Korea; ^6^ Department of Mechanical and Nuclear Engineering Khalifa University of Science and Technology Abu Dhabi 127788 UAE; ^7^ Department of Mechanical Engineering Korea Advanced Institute of Science and Technology (KAIST) Daejeon 34141 Republic of Korea; ^8^ SanaHeal Inc. Cambridge MA 02143 USA; ^9^ School of Transdisciplinary Innovations Seoul National University Seoul 08826 Republic of Korea; ^10^ Interdisciplinary Program in Bioengineering College of Engineering Seoul National University Seoul 08826 Republic of Korea; ^11^ Department of Transdisciplinary Medicine Seoul National University Hospital Seoul 03080 Republic of Korea

**Keywords:** fibers, hydrogels, implantable bioelectronics, multimodal, neural interfaces

## Abstract

Hydrogels have emerged as promising materials for anti‐fibrotic neural interfaces due to their mechanical and chemical similarity to biological tissue. However, their use in multimodal platforms remains limited, owing to fabrication challenges in microstructuring multiple functional hydrogels into compact architectures. Here, a hydrogel thermal drawing process (HG‐TDP) is presented that enables the co‐fabrication of multiple thermoplastically deformable hydrogels into a single, compact, and multifunctional fiber. By optimizing key process parameters, all‐hydrogel neural interfaces are developed that minimize gliosis through tissue‐like mechanical compliance and enable post‐implantation anti‐inflammatory drug delivery via the hydrogel‐based matrix. These fibers feature the compact integration of diverse hydrogel components, including a step‐index optical waveguide, an electrically conductive hydrogel electrode, and a hydrogel‐based microfluidic channel within a unified fiber structure. This integration enables multimodal neural interfacing, as demonstrated by high‐quality neural signal recording, optogenetic stimulation, and localized chemical modulation of neural circuits. This work offers a scalable route toward compact, fully hydrogel‐based neural interfaces that combine multimodal functionality with tissue‐friendly, anti‐fibrotic properties.

## Graphical abstract

Hydrogels demonstrate material properties that mimic the mechanical and chemical environments of biological tissues. Yet, they face challenges during their integration into 3D interfaces. By identifying a class of thermoplastic hydrogels, a strategy is developed to pattern hydrogels in thermally drawn fibers. Composed of functional hydrogels, these fibers act as a platform for multimodal interfacing and antifibrotic interaction with neural tissue.

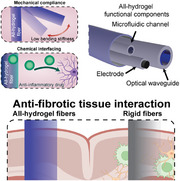

## 1 Introduction

Hydrogels have emerged as ideal materials for implantable electronics due to their mechanical and chemical similarity to biological tissues. Their tissue‐level mechanical compliance, with Young's modulus ranging from the kilopascal to megapascal scale, allows them to accommodate micromotions in the brain caused by respiration and blood circulation. While other soft materials, such as bottle‐brush polymers and soft elastomers, can also replicate tissue‐level stiffness, hydrogels uniquely offer biochemical affinity, including molecular permeability and biochemical versatility, making them particularly well‐suited for anti‐fibrotic neural interfaces.^[^ ^1^, ^2^, ^3^, ^4^ ^]^ Moreover, hydrogels can function as reservoirs for water‐soluble drugs and facilitate molecular‐level interactions with surrounding tissue.

Integrating hydrogels into multimodal neural interfaces presents promising opportunities for seamless bioelectronic‐tissue integration. However, most existing hydrogel‐based systems are limited to single modalities, such as optical, electrical, pharmacological, or mechanical interfacing.^[^ ^5^, ^6^, ^7^, ^8^, ^9^, ^10^, ^11^, ^12^, ^13^, ^14^, ^15^, ^16^ ^]^ These unimodal platforms face inherent challenges in supporting simultaneous sensing and actuation, which are crucial for multifunctional neural systems.^[^ ^17^ ^]^ Multimodal neural interfaces demand coordinated electrical, optical, and chemical access to neural tissue to probe and modulate complex brain functions.^[^ ^18^, ^19^, ^20^, ^21^ ^]^ Although several multimodal hydrogel systems have been explored through mechanical assembly techniques, they often lack the spatial precision and integration density needed to compactly incorporate multiple functional hydrogels.^[^ ^22^ ^]^ This limitation constrains both the modality diversity and scalability of hydrogel interfaces. Thus, developing design strategies for the precise integration of multiple functional hydrogels is essential for advancing compact, multimodal hydrogel‐based neural interfaces.

The thermal drawing process is a well‐established technique for fabricating multimaterial and multifunctional interfaces.^[^ ^23^, ^24^ ^]^ Materials such as elastomers, liquid metals, and various polymers have been successfully integrated into complex geometries through thermal drawing, enabled by matching their rheological properties at elevated temperatures.^[^ ^25^, ^26^, ^27^ ^]^ However, hydrogels typically exhibit poor compatibility with high‐temperature processes, often leading to bubble formation, thermal degradation, and fiber breakage.

In this work, we introduce a hydrogel thermal drawing process (HG‐TDP) to overcome these limitations and unlock new capabilities for multimodal hydrogel interfacing. By identifying a class of thermoplastic polyurethane hydrogels that exhibit favorable dry‐state thermal processability, we establish a scalable fabrication strategy for compact, all‐hydrogel fibers that integrate multiple functional modalities. These fibers incorporate three key functions within a single structure: optical stimulation via a step‐index hydrogel waveguide, electrophysiological recording via a conductive hydrogel electrode, and localized drug delivery through an embedded microfluidic channel. The compact configuration enables simultaneous neural monitoring and modulation, including single‐unit recordings, optogenetically evoked potentials, and chemically induced neural activation. Furthermore, the large‐area release of anti‐inflammatory drugs directly from the hydrogel matrix mitigates glial scarring at the interface, promoting anti‐fibrotic implantation. Finally, we validate the utility of our platform in freely moving mice by demonstrating multimodal recordings and stimulations in behavioral neuroscience settings, including optogenetic modulation and chemically induced seizures with concurrent electrophysiological monitoring.

## 2 Results

### 2.1 Design of Compact, Multifunctional, Anti‐Fibrotic All‐Hydrogel Fibers

**Figure 1:**
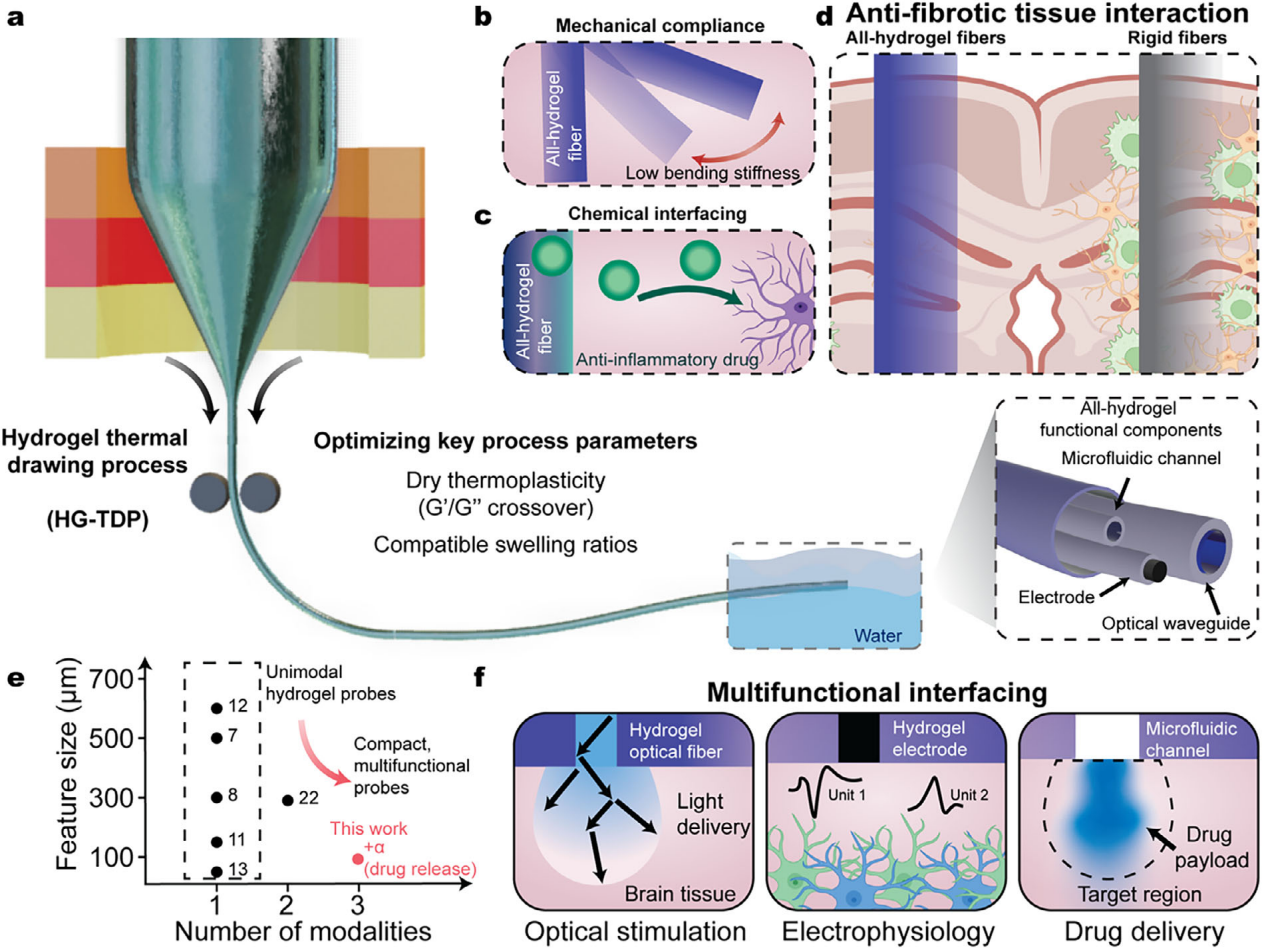
Design of compact, multifunctional, anti‐fibrotic all‐hydrogel fibers. a) Schematic of the HG‐TDP, enabling single‐step fabrication of multifunctional hydrogel fibers. b–d) Schematic showing biochemical (b) and mechanical (c) interactions of all‐hydrogel fibers with neural tissue, leading to reduced foreign body response (d). e) Comparative plot of existing hydrogel‐based neural interfaces by the number of modalities and feature size. Numbers indicate key reference numbers. f) Illustration of integrated functionalities from the tip of the fiber: hydrogel electrode, optical waveguide, and microfluidic channel.

To achieve compact integration of multifunctional components within a soft, fully biocompatible architecture, we developed an all‐hydrogel fiber using the HG‐TDP (**Figure** **1**a). Key material criteria for the integration of hydrogels were identified, including rheological properties and swelling ratios, to enable compact multimaterial hydrogel interfaces. By fabricating both the functional components (i.e., electrode, optical waveguide, and microfluidic channel) and the bulk of the fiber entirely from hydrogels, the resulting mechanical compliance more closely matches that of brain tissue, surpassing that of hybrid hydrogel interfaces (Figure 1b). Furthermore, with hydrogels forming the outermost layer of the fiber, large‐area biochemical interactions with neural tissue are enabled, such as the sustained release of loaded anti‐inflammatory drugs (Figure 1c). This combination of mechanical compliance and chemical interfacing promotes anti‐fibrotic outcomes by minimizing tissue damage and attenuating the acute immune response (Figure 1d).

In addition to providing a platform for anti‐fibrotic interfaces, this scalable fabrication strategy allows for the simultaneous formation of multiple functional modules through a single‐step thermal drawing process. Our device exhibits a unique combination of high functional density and miniaturized cross‐sectional dimensions compared to previously reported hydrogel‐based neural interfaces (Figure 1e). The resulting all‐hydrogel fiber integrates three essential functionalities for neural interfacing within a single, compact structure: optical stimulation via a step‐index hydrogel waveguide, electrophysiological recording through conductive hydrogel electrode, and localized drug delivery using an embedded microfluidic channel (Figure 1f).

### 2.2 Material Design and Characterization for Thermal Drawing of All‐Hydrogel Fibers

To realize HG‐TDP, we first identified key material requirements necessary for compatibility with the thermal drawing process. Materials should exhibit thermoplastic behavior while maintaining stability under prolonged heating (>30 min) and retaining mechanical integrity.^[^ ^28^ ^]^ However, many commonly used hydrogels are chemically crosslinked thermosets, making them inherently incompatible with this process. Additionally, hydrated hydrogels contain water that rapidly evaporates under heat, causing mechanical instability and fiber breakage.

**Figure 2:**
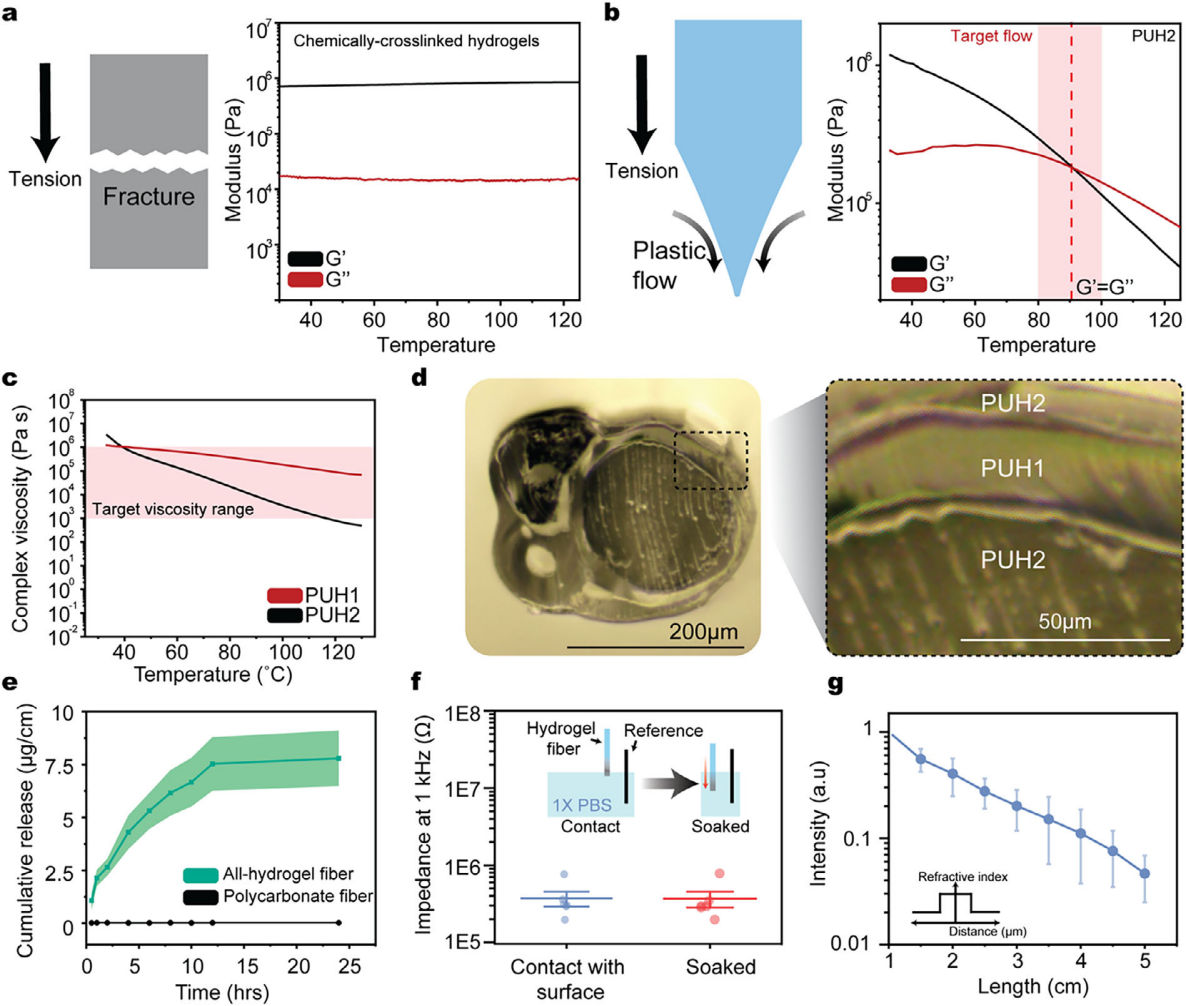
Material design and fabrication strategy for multifunctional all‐hydrogel fibers. a,b) Temperature‐dependent rheological profiles (storage modulus G′ and loss modulus G″) of (a) conventional dry hydrogels and (b) thermoplastic hydrogels. c) Complex viscosity of thermoplastic hydrogels (PUH1 and PUH2) as a function of temperature. The optimal viscosity range for thermal drawing is marked in red. d) Full image (left) and zoomed‐in image (right) of the all‐hydrogel fiber cross‐section. Zoomed‐in image demonstrates a 20µm thick, concentric layer of PUH1. e) Cumulative release of small molecules from hydrogel fibers compared to polymer‐based fibers (*n* = 6). f) Impedance of hydrogel fibers measured at the tip and after exposure of 1 cm length to saline (*n* = 5). g) Normalized optical intensity of hydrogel fibers versus length (*n* = 6). All data in e–g are presented as mean ± standard deviation.

To address these challenges, we identified a class of dry‐processable thermoplastic hydrogels, polyurethane hydrogels (PUH1 and PUH2), that meet the thermal and mechanical requirements for HG‐TDP (Figures S1 and S2, Supporting Information). Unlike single‐monomer hydrogels, these materials are based on hydrophilic polyurethane polymers featuring soft and hard segment domains. Conventional chemically crosslinked hydrogels, such as dry polyethylene glycol diacrylate (PEGDA) hydrogels, do not demonstrate liquid‐to‐solid transitions (i.e., G′/G″ crossover) due to their thermoset nature and constrained polymer networks, thus preventing their integration into the thermal drawing process (**Figure** **2**a). In contrast, polyurethane hydrogels display distinct liquid‐to‐solid transitions, indicative of thermoplastic behavior suitable for thermal processing (Figure 2b).^[^ ^27^ ^]^ Moreover, their complex viscosities (10^3^–10^6^ Pa·s) are sufficiently high to preserve fiber geometry and prevent breaking during thermal drawing (Figure 2c; Figure S2, Supporting Information).^[^ ^27^, ^29^ ^]^ The complex viscosities of PUH1 and PUH2 (10^5^ and 10^4^ Pa·s in the thermal drawing temperature range 85 °C) were critical for preventing collapse of the fluidic channel during the thermal drawing process. PUH1 and PUH2 demonstrate distinct material properties stemming from differences in chemical composition. PUH1 exhibits higher ether content as evaluated by the ether area ratio from Fourier‐transform infrared spectroscopy (PUH1 for 5.07 ± 0.01 (*n* = 3) and PUH2 for 3.76 ± 0.01 (*n* = 3) normalized to the CH_2_ peak, Figure S3, Supporting Information).

The polyurethane hydrogels (PUH1 and PUH2, 52% and 42% water content and 110% and 70% swelling ratios respectively) also exhibit comparable swelling behavior (within 10 wt.% water content and 40% swelling ratios of each other; Figure S4, Supporting Information), which is critical for minimizing delamination or distortion in multimaterial fibers. Upon hydration, both materials show soft mechanical properties appropriate for implantation, with Young's moduli of 2.9 and 39.2 MPa, respectively (Figures S5–S6, Supporting Information).

By integrating hydrogels into the thermal drawing process, the polyurethane hydrogels can be spatially organized into concentric structures with individual layer thicknesses down to ≈20µm (Figure 2d). Due to differences in their refractive indices after swelling, arising from distinct water contents, PUH1 and PUH2 are well‐suited for use as core and cladding materials in step‐index hydrogel optical fibers (Table S1, Supporting Information). In parallel, we developed an electrically conductive thermoplastic hydrogel (ECTH) by blending reduced graphene oxide (rGO) and poly(3,4‐ethylenedioxythiophene): poly(styrene sulfonate) (PEDOT: PSS). The ETCH electrode exhibits both low electrical impedance and complex viscosity compatible with HG‐TDP (Figures S7 and S8, Supporting Information). The integrated ETCH electrode demonstrates comparable mechanical properties with the pristine hydrogel matrix (storage modulus of dry PUH2 is 0.9 MPa; ETCH electrode is 4 MPa at 37 °C and 0.1 Hz shear frequency), minimizing effect on the mechanical properties of the multifunctional all‐hydrogel fiber. The spatial arrangement of the individual functional hydrogels is designed via separate preforms and integrated into a multifunctional all‐hydrogel preform before the thermal drawing process (Figure S9, Supporting Information).

Following the successful integration of these functional hydrogels into a compact fiber architecture, we evaluated the performance of the resulting multimodal hydrogel interface. In the final device, the all‐hydrogel fiber demonstrates a water content of 46 wt.% and a swelling ratio of 90 wt.%, comparable to widely utilized hydrogels such as poly(2‐hydroxyethyl methacrylate) hydrogels.^[^ ^30^ ^]^ The all‐hydrogel fibers possess three key modalities: drug delivery, electrical interfacing, and optical stimulation, demonstrating improved multimodality in comparison with previously reported hydrogel neural interfaces (Table S2, Supporting Information). The all‐hydrogel fiber demonstrates two independent drug delivery systems: 1) Microfluidic drug delivery localized at the tip of the fiber and 2) Pre‐implantation loaded large‐area drug delivery along the longitudinal side of the fiber (Figure S10, Supporting Information).^[^ ^31^, ^32^ ^]^ The microfluidic channel enables sufficient fluid delivery at injection speeds for intracortical injection, highlighting its potential for precise viral delivery and chemical modulation of neural tissue (Figure S11, Supporting Information). The pre‐implantation loaded large‐area drug delivery enables the sustained release of small molecules, such as anti‐inflammatory drugs and fluorescent molecules, from the bulk hydrogel matrix over 24 h, outperforming conventional plastics such as polycarbonate, which are limited to surface adsorption (Figure 2e). In addition, the fiber achieves precise interfacing with neural tissue at its tip while exhibiting low electrical impedance, thereby minimizing noise during electrophysiological recording (Figure 2f). The hydrogel waveguide also shows low optical attenuation (Figure 2g), supporting its application for light delivery during optogenetic stimulation.

### 2.3 Anti‐Fibrotic Properties of All‐Hydrogel Neural Interfaces

**Figure 3:**
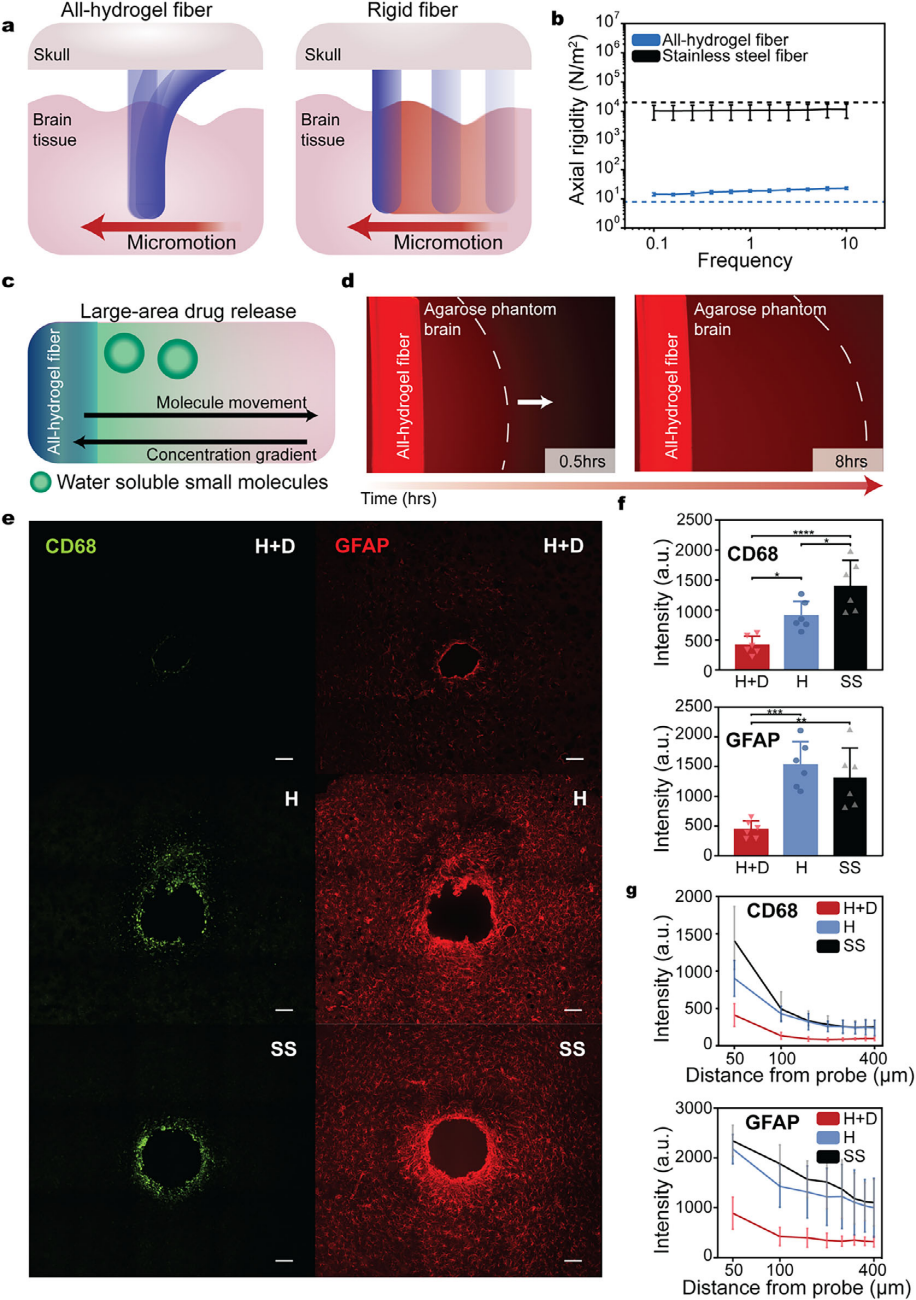
Synergistic mechanochemical interactions for anti‐fibrotic neural interfaces. a) Schematic comparing the compliance of all‐hydrogel fibers and rigid stainless steel wires. b) Axial rigidity of hydrated and dry all‐hydrogel fibers (*n* = 6) versus stainless steel wires (*n* = 3). c) Schematic of large‐area drug release from all‐hydrogel fibers. d) Fluorescence images showing chemical diffusion of rhodamine B from the sidewall of an all‐hydrogel fiber into an agarose phantom brain after 0.5 and 8 h of incubation at room temperature. Dashed lines denote iso‐intensity contours. e) Representative immunohistochemistry images of the implantation sites (parietal cerebral cortex) for stainless steel (SS), all‐hydrogel (H), and dexamethasone‐loaded hydrogel (H+D) fibers after 1 week. Tissues stained with CD68 and GFAP. Scale bar = 100 µm. f) Quantitative analysis of CD68 and GFAP expression surrounding the implanted fiber. g) Spatial intensity profiles of CD68 and GFAP from the probe boundary. One‐way ANOVA with Tukey's post hoc test (*n* = 6, ^*^ *p* < 0.05, ^**^ *p* < 0.005, ^***^ *p* < 0.0005, ^****^ *p* < 0.0001).

We evaluated the anti‐fibrotic potential of all‐hydrogel fibers through a combination of mechanical testing, small‐molecule diffusion studies in tissue‐mimicking models, and in vivo validation. Neural interfaces fabricated from rigid materials, such as metals and silicon, struggle to conform to micromotions associated with neural tissue.^[^ ^33^ ^]^ In contrast, the all‐hydrogel device follows neural tissue dynamics and remains relatively free from micromotion‐induced strain (**Figure** **3**a). To assess the effects of mechanical compliance, we performed dynamic mechanical analysis to characterize the axial stiffness of the hydrogel fibers (Figure 3b). Owing to the low Young's modulus of polyurethane‐based hydrogels, the all‐hydrogel fibers exhibited a three‐order‐of‐magnitude reduction in stiffness compared to stainless steel wires (Figure S12, Supporting Information), indicating significantly enhanced mechanical compatibility with brain tissue. This compliance is expected to reduce micromotion‐induced mechanical stress and subsequent glial activation.^[^ ^34^ ^]^

We further evaluated the fibers’ biochemical interfacing by examining small‐molecule diffusion through the hydrogel matrix. Rhodamine B was loaded along the fiber length and visualized in an agarose‐based brain phantom, confirming sustained and spatially distributed molecular diffusion from the hydrogel surface (Figure 3c). The fiber demonstrated increased retention of rhodamine B with higher loading solution concentrations, suggesting a diffusion‐driven loading mechanism (Figures S13 and S14, Supporting Information).^[^ ^35^ ^]^ Additionally, the fibers exhibited a comparable release profile for dexamethasone sodium phosphate, indicating compatibility with small‐molecule drugs possessing increased ionic character. To characterize the release profile in vivo, the long‐term release profile of the small molecule, Rhodamine B, was measured. Over a 4‐day period, drug release reached equilibrium within 2 days (Figure S15, Supporting Information).

To validate anti‐fibrotic effects in vivo, we implanted the all‐hydrogel fibers into the CA3 region of the mouse hippocampus and assessed host immune response after one week using immunohistochemistry (Figure 3d). Immune activation was quantified by measuring the expression of CD68 (a marker for activated microglia) and GFAP (a marker for reactive astrocytes).

To validate the mechanical anti‐fibrotic effects of the all‐hydrogel fibers in comparison with commercial neural implants, we performed immunohistochemistry of all‐hydrogel fibers and silica optical fibers (Figure S16, Supporting Information). Quantitative analysis showed that hydrogel fibers demonstrate reduced astrocyte expression compared to silica controls, highlighting the contribution of tissue‐like mechanical compliance to immune response mitigation (*P* = 0.02 GFAP; *P* = 0.06 CD68; Figure S17, Supporting Information).

To verify the combined effect of mechanical softness and large‐area chemical interaction, responses were compared across three groups: stainless steel wire (SS), pristine all‐hydrogel fiber (H), and dexamethasone‐loaded hydrogel fiber (H+D). The hydrogel fiber's diffusive drug release profile enabled chemical suppression of the acute immune response immediately following implantation. Dexamethasone sodium phosphate, a widely used anti‐inflammatory agent to target the acute immune response, was selected to mitigate acute tissue damage during device insertion.^[^ ^36^, ^37^ ^]^ Furthermore, the target administration time frame aligns with the delivery time frame of the all‐hydrogel fiber at ≈2 days.^[^ ^38^ ^]^

Quantitative analysis revealed that the all‐hydrogel fiber significantly reduced CD68 expression compared to the stainless‐steel control (*P* = 0.025; Figure 3e), whereas GFAP intensity remained comparable to the control. In contrast, the dexamethasone‐loaded fiber further suppressed immune activation, exhibiting significantly lower expression of both CD68 and GFAP compared to the SS group (*P* < 0.0001, *P* = 0.0025, respectively) and the H group (*P* = 0.028, *P* = 0.0004, respectively) (Figure 3e). Additionally, there was a substantial reduction in the density of activated astrocytes and macrophages within a 100 µm radius of the fiber, which is known to be a region critical for achieving stable electrical interfacing with single neurons (Figure 3f).^[^ ^39^ ^]^ These results suggest that the combination of mechanical compliance and localized anti‐inflammatory drug delivery synergistically mitigates foreign body responses at the tissue–device interface.

### 2.4 Functional Insulation and Multimodal Electrophysiology in All‐Hydrogel Fibers

**Figure 4:**
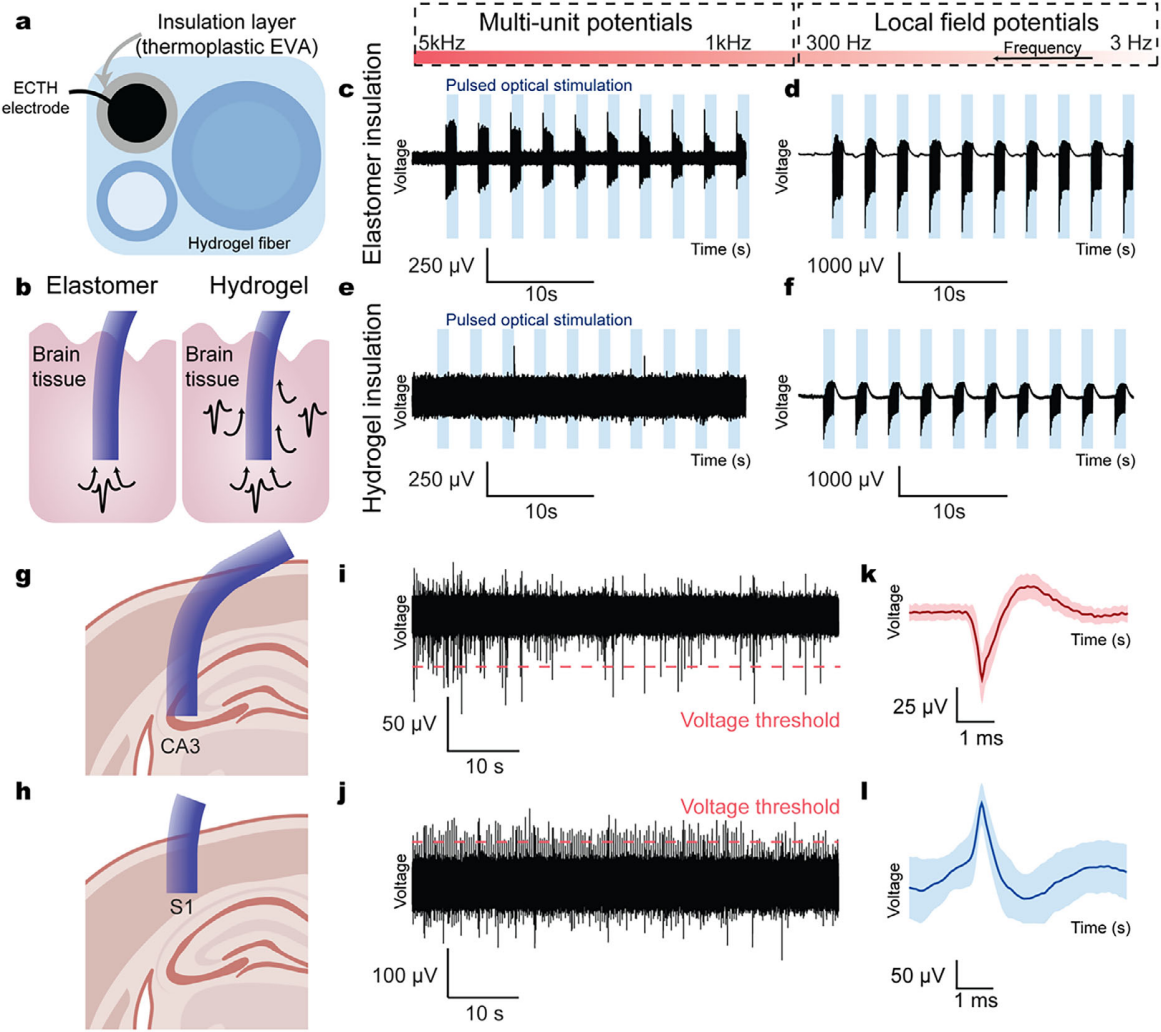
Robust insulation and multimodal signal recording using hydrogel‐elastomer hybrid fibers. a) Schematic of the insulation layer in the all‐hydrogel fiber. b) Schematic indicating improved signal quality from insulated hydrogel fibers compared to uninsulated counterparts. c,d) Optically‐evoked (c) multi‐unit potentials and (d) local field potentials recorded using hydrogel fibers insulated with thermoplastic EVA. e,f) Optically‐evoked (e) multi‐unit potentials and (f) local field potentials recorded using uninsulated hydrogel fibers. g,h) Schematic of all‐hydrogel fiber recording single‐unit potentials from the (g) hippocampus and (h) somatosensory cortex. i,j) Representative single‐unit spike traces recorded using all‐hydrogel fibers in vivo of (i) negative polarity spikes and (j) positive polarity spikes. k,l) Averaged waveforms of thresholded (k) negative polarity spikes and (l) positive polarity spikes.

The HG‐TDP platform offers precise spatial control and multimaterial integration, enabling the incorporation of thin insulating layers within all‐hydrogel fibers. Through HG‐TDP, we integrated a thin elastomeric insulator layer, ethylene‐vinyl acetate (EVA), directly between the conductive electrode and the surrounding bulk hydrogel to ensure robust electrical insulation (**Figure** **4**a). This soft insulator (approximately Young's modulus 3 MPa, 40% vinyl acetate content) demonstrates similar mechanical properties to the thermoplastic polyurethane hydrogels, minimizing its effect on the mechanical properties of the integrated multifunctional fiber.^[^ ^40^ ^]^ Furthermore, due to its thermoplastic properties, EVA can be co‐drawn with other hydrogel components and allows the fabrication of electrically insulated and miniaturized hydrogel electrodes embedded in a hydrogel matrix. We demonstrate this miniaturization enables improved resolution in electrophysiological recordings (Figure 4b).

Combined with the embedded optical waveguide, these fibers reliably recorded high signal‐to‐noise ratio (SNR) optically‐evoked potentials in Thy1‐ChR2 transgenic mice in both local field potentials and multi‐unit activities across multiple stimulation trains, as seen in representative stimulation waveforms (Figure 4c,d; Figure S18, Supporting Information). In comparison, uninsulated fibers did not record high‐frequency multi‐unit activities, while evoked potentials in local field potentials were observed (Figure 4e,f; Figure S18, Supporting Information). In addition, EVA‐insulated all‐hydrogel fibers maintain robust recording quality across a range of optical stimulation frequencies (Figures S19 and S20, Supporting Information). Neural responses attenuated above 30 Hz, consistent with the known kinetics of Channelrhodopsin‐2 activation, supporting the biological origin of the recorded signals (Figure S21, Supporting Information).^[^ ^41^, ^42^ ^]^ To further confirm that the potentials are optically evoked, the temporal dynamics of the evoked waveforms were evaluated (Figure S22, Supporting Information). Stimulation latency between the onset of optical stimulation and the negative peak of the evoked potential demonstrates a median latency of 3.4 ms. The latency is consistent with the 2 to 6 ms latency of Channelrhodopsin‐2 and demonstrates no latency consistent with light‐evoked artifacts (<1 ms).^[^ ^41^, ^43^, ^44^ ^]^

The high‐fidelity insulation also facilitated the detection of low‐amplitude endogenous neural signals in wild‐type mice in both the hippocampal CA3 region and primary somatosensory cortex (S1) (Figure 4g,h). The fibers successfully recorded both negative‐ and positive‐polarity (3 Hz firing rate, 517 detected spikes) extracellular single‐unit activities from the CA3 region and S1 region, respectively, confirming their suitability for precise in vivo electrophysiological recording (Figure 4i–l; Figure S23, Supporting Information).

To evaluate the long‐term stability of the all‐hydrogel fibers, the fibers were incubated in environments mimicking physiological conditions (37 °C in saline solution).^[^ ^45^ ^]^ Optical fibers demonstrated stable optical output across 3 weeks (≈70% of baseline output) and power density sufficient for optogenetic stimulation in vivo (Figure S24, Supporting Information).^[^ ^46^ ^]^ Furthermore, the electrode also demonstrates stable impedance (within 50% of baseline impedance) and raw impedance values suitable for intracortical neural interfaces (≈1MΩ at 1kHz), demonstrating electrical properties for long‐term electrophysiology (Figure S25, Supporting Information).^[^ ^47^ ^]^ Thus, the all‐hydrogel fibers demonstrate a level of resistance to hydrolysis during long‐term implantation.

Chronic electrophysiology of local field potentials in the hippocampus was conducted with ETCH electrodes from all‐hydrogel fibers. The ETCH electrodes demonstrate similar amplitude waveforms at 1 week and 3 weeks after implantation (Figure S26, Supporting Information).

### 2.5 Multimodal Applications of All‐Hydrogel Fibers in Freely Moving Mice

**Figure 5:**
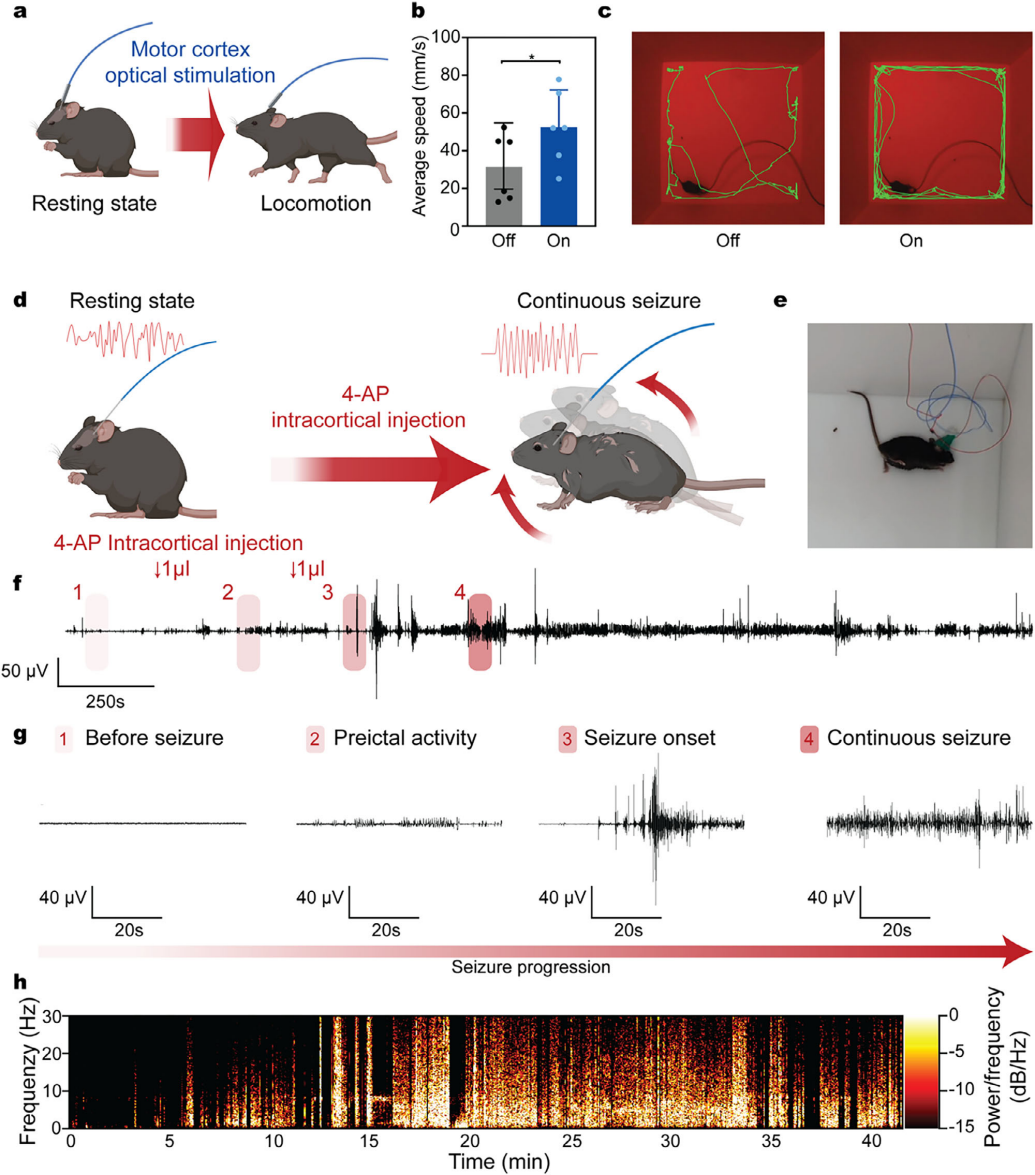
In vivo application of multimodal all‐hydrogel fibers in freely moving mice. a) Schematic of optogenetic stimulation targeting the M2 to modulate locomotor activity. b) Average locomotion speed with stimulation ON versus OFF (*P* = 0.04). Values represent the mean and standard deviation (number of animals, *n* = 6, ^*^ *p* < 0.05; One‐sided t‐test). c) Representative trajectory plots of mouse movement under spontaneous (OFF) and optogenetic (ON) conditions. d) Schematic of seizure induction via intracortical injection of 4‐AP through the fiber's microfluidic channel, with simultaneous electrophysiological recording. e) Photo of a mouse exhibiting seizure behavior. f) Electrophysiological traces from the CA3 hippocampus showing resting, preictal, seizure onset, and ictal states. Two 1µl infusions of 4‐AP are conducted at the marked arrows. g) Time‐aligned electrophysiological traces illustrating the transition across the four distinct seizure states. h) Spectrogram showing increased power in the 10–30 Hz band during the ictal phase during seizure progression.

The compact and lightweight design of the all‐hydrogel fiber enables its use in freely moving mice, with implanted devices light enough to avoid disrupting natural behaviors. To assess functional performance in behavioral contexts, we implanted the fiber into the secondary motor cortex (M2), a region involved in regulating locomotion and motor control (**Figure** **5**a).^[^ ^48^ ^]^ During optogenetic stimulation, mice exhibited significantly increased locomotor activity compared to baseline spontaneous behavior. The average running speed under stimulation was markedly higher (*P* = 0.04), indicating that the hydrogel‐based optical interface effectively modulates M2‐mediated motor function (Figure 5b,c).

To further demonstrate multimodal capability, we performed localized chemical delivery and simultaneous neural recording to investigate seizure‐like activity in freely moving animals (Figure 5d; Figure S27, Supporting Information). Following implantation into the CA3 region of the hippocampus, focal seizures were induced by intracortical infusion of 4‐aminopyridine (4‐AP) through the embedded microfluidic channel while recording electrophysiological signals in real time.^[^ ^49^ ^]^

The 4‐AP injection elicited characteristic seizure‐like behaviors, accompanied by epileptiform waveforms in the recorded signals (Figure 5e,f). Seizure amplitudes increased over time, progressing through distinct stages from resting activity to continuous seizure waveforms (Figure 5g). Spectrogram analysis revealed increased neural activity within the 10–30 Hz band during the ictal phase, consistent with known signatures of 4‐AP‐induced seizures (Figure 5h).^[^ ^50^ ^]^ These results confirm that the all‐hydrogel fiber enables multimodal neuromodulation, supporting both localized drug delivery and high‐fidelity signal acquisition during complex neurobehavioral events.

## 3 Discussion and Conclusion

Although hydrogel‐based neural interfaces have been explored in various formats, including surface coatings and hybrid composites with single or dual functionalities, truly multimodal implementations have remained limited (Table S2, Supporting Information).^[^ ^51^ ^]^ In this study, we present an all‐hydrogel fiber platform that integrates three essential functions within a single, compact device: (1) a step‐index waveguide for optical stimulation, (2) conductive hydrogel electrodes for electrophysiological recording, and (3) embedded microfluidic channels for localized drug delivery. This level of functional integration substantially broadens the capabilities of hydrogel‐based interfaces, enabling electrical, optical, and chemical interaction with neural tissue to support comprehensive probing and modulation of neural circuits.

A key advancement of our approach lies in leveraging the intrinsic chemical functionality of hydrogels to deliver bioactive molecules directly from the matrix, enabling localized immune modulation and minimizing glial encapsulation. While the current study is limited to evaluating the immune response in time frames of one week, we predict a reduction in long‐term immune response due to a cascade effect. As microglial activation occurs within the first 24 h, and astrocytic gliosis begins after 48 h, we hypothesize that intercepting these early events will play a critical role in preventing long‐term scarring according to previous literature.^[^ ^37^ ^]^ When combined with tissue‐level mechanical compliance, these anti‐fibrotic effects allow for more stable and long‐term neural interfacing. This represents a significant departure from conventional hydrogel integration techniques, such as 3D printing or dip‐coating, which are generally confined to surface layers or rigid substrates with limited functional complexity.^[^ ^10^, ^22^, ^52^, ^53^ ^]^

The HG‐TDP introduced here enables the co‐fabrication of multiple functional hydrogels and insulating elastomers with microscale precision, offering a scalable and versatile platform for constructing fully integrated, multimodal neural probes. We proposed and validated three key design criteria for hydrogel selection in HG‐TDP: (1) thermoplastic behavior in the dehydrated state, (2) rheological compatibility with thermal drawing conditions, and (3) matched swelling behavior among components. These criteria guided the fabrication of fibers with functional layers as thin as 20 µm, demonstrating fine spatial resolution and structural robustness. The integration of a thin insulating material in the all‐hydrogel fiber demonstrated improved recording in high‐frequency ranges (300 to 5000 Hz). This is seen to be due to the large effective electrode surface area in the uninsulated hydrogel fiber and effective low‐pass filtering.^[^ ^54^ ^]^ We demonstrate that the integration of functional hydrogel interfaces embedded with electrical insulation materials remains a design factor in the fabrication of all‐hydrogel interfaces.

By overcoming longstanding limitations in hydrogel integration, thermal processability, material compatibility, and functional diversity, HG‐TDP offers a generalizable platform for constructing next‐generation hydrogel‐based bioelectronic interfaces. While in this study, 4‐AP was used as a proof‐of‐concept study to validate concurrent delivery and electrophysiology, future work would apply the hydrogel fiber platform for localized seizure suppression with localized delivery of seizure suppression agent, such as GABA. Such platforms have the potential to support closed‐loop control systems in neuroscience, enable long‐term implantation with minimal immune response, and accelerate the development of soft bioelectronics for both basic research and preclinical applications.

## 4 Experimental Section

### Fabrication of All‐Hydrogel Fibers and Chemically Cross‐Linked Hydrogels

To fabricate the all‐hydrogel fibers, a macroscopic preform was initially prepared. PUH1 and PUH2 were HydroMed D4 and D7 (AdvanSource Biomaterials), respectively. Thermoplastic PUH2 was first cast into a block via molding and subsequently shaped into a cylindrical core by rolling on a hot plate. For the cladding of the hydrogel optical waveguide, PUH1 was hot‐pressed into sheet form and then wrapped around the cylindrical PUH2 core, creating a step‐index waveguide structure. The hydrogel electrode was fabricated from a composite of rGO (Sigma–Aldrich) and PEDOT: PSS (PH1000, Clevios). The electrode preparation involved dissolving PUH2 in an 85% ethanol solution, which was then mixed thoroughly with the rGO suspension and PEDOT: PSS. The mixture was solvent‐casted into sheets, which were subsequently rolled to form hydrogel electrode rods. A microfluidic channel was fabricated by creating a hollow structure enclosed by an outer layer of PUH2. Upon completing the preparation of all functional components—namely, the hydrogel waveguide, hydrogel electrode, and hydrogel microfluidic channel—these were collectively wrapped with additional sheets of PUH2.

The thermal drawing process was conducted at preform feed rates of 5 to 10 mm min^−1^ and fiber draw speed ranging from 2 to 8 m min^−1^. A three‐temperature zone furnace (Mellen PS205 series) was utilized with zone 1 set to 60 to 70 °C, zone 2 set to 85 to 100 °C and zone 3 set to 60 to 70 °C. The final device consists of a single 400 µm outer diameter fiber (device size range 300 to 600 µm) that integrates (i) a step‐index optical waveguide, (ii) a conductive hydrogel electrode, and (iii) a microfluidic channel. PEGDA hydrogels were synthesized by creating a mixture of 20 w/v% PEGDA (M_n_ 700, Sigma–Aldrich) in deionized water and mixed with 2‐2‐hydroxy‐4′‐(2‐hydroxyethoxy)‐2‐methylpropiophenone (Irgacure 2959, Sigma–Aldrich) to create a 1 w/v% ratio (photoinitiator to monomer). The mixture was cured with ultraviolet (UV) light for 4 min.

### Characterization of Multifunctional Hydrogel Neural Probe

Electrochemical impedance spectroscopy was conducted using a potentiostat (Biologic, SP‐300) or LCR meter (Hioki, IM3590) to evaluate both the impedance characteristics of the hydrogel electrode and the electrical insulation performance of the hydrogel encapsulation. Impedance measurements were performed with fibers at 1cm in length. 10mV were applied at the hydrogel electrode, and frequency sweeps were conducted from 1 Hz to 100kHz. Impedance was measured at 100mV alternating voltage (Good Will Instrument Co., Ltd., LCR‐6100) during in vitro tracking. Samples were incubated in a saline bath at 37 °C.

Optical transmission properties of the hydrogel optical waveguide were assessed using a 465 nm laser source (IOS‐465, RWD) and a power meter (S121C and PM100D, Thorlabs). To determine attenuation loss, hydrogel fibers were sequentially shortened from an initial length of 5 cm to shorter lengths in 0.5 cm increments, with transmitted light intensity measured at each length. During in vitro tracking, samples were cut to a 5 mm length and measured. Samples were incubated in a saline bath at 37 °C between measurements.

The infusion dynamics of the hydrogel microfluidic channel were systematically characterized via injection through a microinjection system and microsyringe (Hamilton 1700 series microsyringe) loaded with deionized water. A 10 µl volume of deionized water was injected through the hydrogel fiber, and the mass of the outputted solution was weighed. The percentage of the solution output mass in relation to the input volume was reported as the return rate. Return rates across a range of injection speeds utilized in intracortical virus injection (10–100 nL s^−1^).

To evaluate diffusion profiles, hydrogel fibers were loaded with target small molecule drugs: rhodamine B (Sigma–Aldrich) and dexamethasone sodium phosphate (Sigma–Aldrich) through incubation in target molecules dispersed in deionized water. The loaded fibers were incubated in deionized water, and 150µl of the incubated solution was sampled and refilled at every time point under gentle shaking. The concentration of released molecules was determined via solution absorbance with a UV–vis spectrometer (SpectraMax M2) at 242 and 550nm for dexamethasone sodium phosphate and rhodamine B, respectively. Hydrogel fibers of length 30 and 5 mm were used for dexamethasone sodium phosphate and rhodamine B, respectively. To account for fiber length, molecule release was reported in µg cm^−1^.

To evaluate tissue diffusion profiles, the fluorescent all‐hydrogel fibers were incubated in a 200 µm rhodamine B bath for 24 h. The edges of the fluorescent all‐hydrogel fibers were secured to the bottom of a petri dish with ultraviolet resin, and a 0.6 wt.% agarose solution prepared in deionized water was poured onto the fiber until immersed. After gelation, images were taken with an inverted fluorescent microscope at 0.5 and 8 h. To visualize the tissue diffusion profiles, isointensity lines were obtained with analysis in ImageJ.

### Characterization of Hydrogels

Rheological characterization was conducted with a rheometer (Anton Paar, MCR 302e) with an 8 mm probe. Temperature sweeps were conducted from 130 to 30 at 5 °C min^−1^, 1% strain. Frequency sweeps were conducted from 0.1 to 100 Hz at 1% strain and 0.1% strain for PUH1 and PUH2, respectively. Axial rigidity measurements were conducted with a dynamic mechanical analyzer (Anton Paar 702e). Hydrated hydrogel fibers of length 10 mm were subject to axial strain 1% at frequencies of 0.1 to 10 Hz. Testing was conducted in comparison with AISI 316L stainless steel fibers (Goodfellow materials, FF215135). Fourier transform infrared spectroscopy (Thermo Fisher Scientific Instrument, Nicolet iS50) was scanned from 400 to 4000 cm^−1^.

### Animal Experiments

All animal experiments were conducted following the guidelines and approval of the Institutional Animal Care and Use Committee (IACUC) at KAIST and Seoul National University. All surgical procedures were performed under sterile conditions to minimize infection risk.

### In vivo Electrophysiology and Optogenetic Stimulation

For in vivo electrophysiological recordings, C57BL/6N mice aged 12–14 weeks (Koatech, South Korea) and B6.Cg‐Tg(Thy1‐COP4/EYFP)18Gfng/J (Thy1‐ChR2‐YFP; Jackson Laboratory) mice were employed for single‐unit and optogenetically evoked potential recordings, respectively. Mice were anesthetized with isoflurane (2–5% in oxygen) delivered through a vaporizer to maintain stable anesthesia. The animals were securely fixed in a stereotaxic frame (RWD Life Science), and a small craniotomy was performed over the target brain region. Insulated hydrogel neural probes were stereotaxically implanted into the CA3 region of the hippocampus (coordinates 2.0 mm mediolateral (ML), −1.7 mm anteroposterior (AP), and −2.1 mm dorsoventral (DV) relative to bregma) and the S1 region (coordinates 2.0 mm ML, −1.7 mm AP, and −1.0 mm DV relative to bregma) for the recording of single‐unit potentials. The mouse was anesthetized with an inhalable anesthesia, isoflurane, and an electrophysiological recording was conducted for 5 min. During the recording, optically‐evoked potentials and long‐term recording, insulated hydrogel neural probes were implanted into the CA3 region of the hippocampus (coordinates 2.0 mm ML, −1.7 mm AP, and −2.1 mm DV relative to bregma). Uninsulated hydrogel neural probes were implanted at (coordinates 3.0 mm ML, −2.8 mm AP, and 1.5 mm DV relative to bregma). Neural signals were recorded using a LABRAT recording system (Tucker‐Davis Technologies). Electrophysiology was conducted in a single‐ended configuration. A separated reference/ground electrode was implanted in the contralateral frontal lobe. The ground electrode was also connected to a Faraday cage. Optogenetic stimulation was conducted at various frequencies with a blue laser 465 nm laser (RWD Life Science, IOS‐465 Intelligent Optogenetics System). Stimulation was conducted with 1 s duration pulse trains (pulse widths 5 ms to 10 ms), 2 s delay between pulse trains, and optical output power below 12 mW from the fiber tip. Optically‐evoked multi‐unit potentials and single‐unit potentials were acquired with analog filtering within the frequency range of 300–5000 Hz, while local field potentials were recorded within the range of 3–500 Hz. Spike detection and average waveforms were obtained using custom Python and MATLAB code. Spiking rate and principal component analysis were conducted on the Offline Sorter (Plexon). Spikes were thresholded in the Offline Sorter with a 5 standard deviation amplitude threshold. High‐amplitude artifacts and unsorted spikes were removed during analysis. The firing rate was obtained by taking the median of the interspike‐interval histogram. Long‐term tracking of local field potentials was acquired with analog filtering of 1 Hz to 1kHz and digitally filtered from 4 to 100 Hz.

### Immunohistochemistry

Immunohistochemical (IHC) analysis to evaluate the foreign body response was performed by labeling for GFAP and CD68. C57BL/6N mice aged 8–10 weeks were utilized for immunohistochemical analysis. Three distinct fiber types—hydrogel neural probes, hydrogel neural probes loaded with dexamethasone, and stainless‐steel fibers—were implanted into the parietal cerebral cortex. Prior to implantation, all fibers underwent sterilization via overnight UV illumination. Additionally, hydrogel neural probes intended for drug delivery were incubated in a dexamethasone solution to facilitate drug loading. After one week of implantation, mice were transcardially perfused first with phosphate‐buffered saline (PBS) followed by 4% paraformaldehyde (PFA) solution. Brains were harvested and post‐fixed overnight in 4% PFA, subsequently immersed in a 30% sucrose solution for cryoprotection. Brain tissues were then sectioned horizontally at a thickness of 40 µm using a cryotome (Leica). Tissue sections were blocked and permeabilized in 0.3% Triton X‐100 (Sigma) and 5% normal donkey serum (Abcam) in PBS. After rinsing in PBS, sections were incubated overnight at 4 °C with primary antibodies: anti‐GFAP (ab53554, Abcam) and anti‐CD68 (ab125212, Abcam). Following primary antibody incubation, sections were fluorescently labeled for 2 h with secondary antibodies: Donkey anti‐goat IgG H&L (Alexa Fluor 594, ab150132) and donkey anti‐rabbit IgG H&L (Alexa Fluor 488, ab150064). Sections were mounted and counterstained with 4′,6‐diamidino‐2‐phenylindole (DAPI) using Vectashield mounting medium (Vector Laboratories). Immunohistochemical images were acquired using a laser‐scanning confocal microscope (Nikon C2 or ZEISS LSM980). Fluorescence intensity was quantified and analyzed using ImageJ software and custom MATLAB scripts.

### Optogenetic Motor Cortex Modulation

Thy1‐ChR2‐YFP mice aged 10–12 weeks underwent implantation of hydrogel neural probes targeting the secondary motor cortex (coordinates: 2.5 mm AP, 1 mm ML, −1.5 mm DV relative to bregma). After a recovery period of one week post‐surgery, mice were individually placed into an open‐field test apparatus in a sound‐attenuated room. Optogenetic stimulation was delivered using a 465 nm laser system (RWD life science). The experimental protocol comprised two distinct epochs: an initial 3‐min baseline period without stimulation, followed by a subsequent 3‐min period of continuous optical stimulation. Stimulation parameters were set at a frequency of 20 Hz with a pulse width of 5 ms. Throughout the procedure, video recordings captured the behavior and positional dynamics of each mouse. Behavioral data were analyzed using ToxTrac software.

### Seizure Induction and Monitoring

C57BL/6N mice aged 8–10 weeks underwent surgical implantation of hydrogel neural probes targeting the CA3 region of the hippocampus. Following a one‐week post‐operative recovery period to ensure optimal healing and stabilization, mice were individually placed in an OFT apparatus enclosed within a Faraday cage to minimize electromagnetic interference. To induce seizure activity, 4‐AP was administered through the hydrogel neural probe using a precision microsyringe pump system (WPI, Nanoliter 2000 injector and Micro4 controller). Two sequential infusions of 4‐AP (45 mm, 1µL each) were delivered via the microfluidic channel at 100 nL min^−1^ with 10‐min intervals, following established focal hippocampal seizure protocols.^[^ ^55^ ^]^ Concurrently, electrophysiological monitoring and recording of neural activity were performed continuously to capture seizure dynamics in real‐time. Electrophysiological recordings obtained during ictal periods were subsequently analyzed with spectrogram analysis. To verify seizure induction, seizure‐like behavior was observed, such as loss of postural tone and the onset of clonic movements. Seizure stages were classified according to the following standards: (i) baseline (endogenous neural signal before 4‐AP infusion), (ii) pre‐ictal activity, defined by transient bursts >2Hz frequency, (iii) seizure onset, activity above 2 Hz with signal amplitudes greater than 3 times the baseline voltage that last for at least 30 s, and (iv) continuous seizure, sustained seizure activity for at least 1 min.^[^ ^56^ ^]^

### Statistical Analysis

All statistical analyses in this study were performed using GraphPad Prism 10 (GraphPad Software, USA). The normality of data distributions for both the M2 behavioral experiments and IHC fluorescence intensity measurements was assessed using the Shapiro‐Wilk test. For the average speed analysis of M2 behavioral experiment, a one‐tailed Student's t‐test was employed to evaluate statistical significance, with a threshold of ^*^ *p* < 0.05. For the quantitative analysis of fluorescence intensity from IHC experiments, an ordinary one‐way analysis of variance (ANOVA) was conducted, followed by Tukey's multiple comparisons test to determine group‐level differences. Significance levels were denoted as follows: ^*^p < 0.05, ^**^p < 0.005, ^***^p < 0.0005, and ^****^p < 0.0001.

## Conflict of Interest

The authors declare no conflict of interest.

## Supporting information



Supporting Information

## Data Availability

The data that support the findings of this study are available from the corresponding author upon reasonable request.;
