# Supplementary material for: Thermally Drawn Multifunctional All‐Hydrogel Fibers for Anti‐Fibrotic and Multimodal Neural Interfaces
Source: Adv Mater. 2025 Oct 9;38(9):e11634. doi: 10.1002/adma.202511634 (PMC12902630; doi:10.1002/adma.202511634)
Supplement: Supplementary file 1 — Supporting Information [file ADMA-38-e11634-s001.docx]

Supporting Information

**Thermally drawn multifunctional all-hydrogel fibers for anti-fibrotic and multimodal neural interfaces**

*Changhoon Sung†, Kum Seok Nam†, Yeji Kim, Honey Kang, Kanghyeon Kim, Chanwoong Yoon, Somin Lee, Ain Chung, Jiheong Kang, Young-Gyun Park, Alan Jung Park, Haider Butt, Hyunwoo Yuk, Seongjun Park**

**Supplementary text**

Preform preparation process

Sheet preparation. As provided PUH1 and PUH2 is dissolved in 85 vol% EtOH:DI solution at 65 to 75˚C overnight. The solution is subsequently mixed until a homogenous solution is achieved. Sheets are solvent cast on Teflon sheets and dried in a convection oven at 65 to 75˚C overnight. Heat consolidation is conducted with a heat gun (Bosch GHG 16-50) unless stated overwise.

Optical waveguide preform fabrication. Custom-design molds were utilized to create large PUH2 blocks. Molds are lined with PTFE sheets to allow easy detachment during removal. As provided PUH2 is loaded into square molds and consolidated into solid PUH2 blocks at 150˚C at -0.1 MPa. The blocks are heated and molded into a cylindrical core. Care is taken not to fold edges of the blocks to prevent the formation of air bubbles. After cooling, PUH1 sheets are wrapped around the PUH2 core to create a step-index waveguide structure and consolidated.

Electrode preform fabrication. 10%w/v PUH2 solutions are prepared in 85 vol% EtOH:DI solution at 65 to 75˚C. Reduce graphene oxide (rGO, Sigma Aldrich 777864) is dispered in a 85 vol% EtOH:DI solution at a concentration of 2.5 mg/ml. PEDOT:PSS (PH1000, Ossila, CAS number 155090-83-8) is filtered through a 0.45 µm syringe filter to remove aggregates before mixing. To prepare the electrode solution, rGO and PEDOT:PSS are added to create a 4 wt% rGO and 0.5 wt% PEDOT:PSS solution in relation to the mass of the PUH2. The electrode solution is dispered in a bath sonicator (Daihan Science, WUC-A02H) for 1 hour. The resulting solution is cast on and dried at room temperature overnight to produce the ETCH sheet. The sheet is folded to create the ETCH and consolidated. An EVA sheet is wrapped around the ETCH for the insulated all-hydrogel fiber while a PUH2 sheet is wrapped around the ETCH for the uninsulated all-hydrogel fiber.

Microfluidic channel preform and multifunctional preform consolidation. PUH2 sheets are wrapped around a 4.5mm PTFE rod and consolidated. Optical waveguide, electrode and microfluidic channel preforms are wrapped with PUH2 sheets and consolidated.

Backend connection of all-hydrogel fibers

Fiber preparation. Before all connection steps, all-hydrogel fibers are swollen in saline or deionized water overnight, until swelling equilibrium is reached.

Optical waveguide connection. All-hydrogel fibers are inserted into a 440µm inner diameter ceramic ferrule (Thorlabs, CF440-10) and swelled. Excess water is removed from the surface of the fiber and is mechanically secured in the ferrule with an epoxy or ultraviolet-curing resin. Excess fiber is trimmed with a Dorco blade.

Microfluidic channel connection. All-hydrogel fibers are inserted into a 5mm to 1cm length of flexible tubing. The space between the all-hydrogel fiber and the flexible tubing is sealed with a UV resin with care taken to ensure that the resin does not cover the end of the fiber.

Electrode connection. For insulated all-hydrogel fibers, the ETCH electrode is mechanically removed from the bulk of the all-hydrogel fiber. Under a microscope, the outer PUH2 layer is physically dissected with a Dorco blade or a 31-gauge needle. The blade is used to cut the surrounding PUH2 layer in the vicinity of the ETCH electrode along the longitudinal length of the fiber. The ETCH electrode is then removed from the bulk of the hydrogel fiber for an approximately 5mm length. Care is taken to ensure that PUH2 layers are separated from the ETCH electrode. ETCH electrodes are dipped in a silver paint and attached to female electrode connectors and dried. The fiber-connector interface is mechanically secured by mechanical coating in a resin layer. For the electrode connection of uninsulated all-hydrogel fibers, backend connection protocols from previous literature is utilized.^[19]^

Multifunctional fiber. One end of the all-hydrogel fiber was cleanly cut with a Dorco blade to expose the microfluidic channel. The hydrogel fiber was inserted into a 5 mm to 1 cm length of flexible tubing and sealed via resin. Resin was placed carefully between the fiber and tubing to prevent overflow and subsequent blockage of the exposed microfluidic channel. After curing, the flexible tubing is connected to a barbed microfluidics connector and microinjection syringe tubing connected to a syringe pump.

To prevent potential tissue occlusion and air bubble formation at the fiber-tissue interface, all-hydrogel microfluidic channels were primed with fluid during in vivo implantation. During recovery, the open end of the flexible tubing was mechanically covered with Blu-Tack to prevent debris from entering. When starting freely moving seizure induction experiments, 4-AP solutions were injected inside the flexible tubing, starting with the end closest to the all-hydrogel fiber to prevent air bubble formation. Barbed microfluidic connectors were subsequently inserted in the flexible tubing to connect to the infusion pump.

|  | Refractive index (De-hydrated) | Refractive index (Hydrated) | Swelling ratio (%) |
| --- | --- | --- | --- |
| Cladding (PUH1) | 1.512 | **1.418** | 110 |
| Core  (PUH2) | 1.527 | **1.453** | 70 |
| ∆ | 0.015 | **0.035** |  |

**Table S1**. Refractive indices and swelling ratios of PUH1 and PUH2 hydrogels in dehydrated and hydrated state. Difference in the refractive index between the two hydrogels increases upon hydration, attributed to their differing swelling ratios.

| **Reference (Application)** | **Softness (Young’s Modulus)** | **Optical waveguide** | **Electrode  (kΩ at 1 kHz)** | **Microfluidic channel** | **Diffusive drug loading** |
| --- | --- | --- | --- | --- | --- |
| All-hydrogel fiber | 2.9 & 39MPa | O (- 3.27 dB/cm) | O (200 kΩ) | O | O |
| 1 (Brain)^[11]^ | Not measured | X | O (≤200 kΩ) | X | X |
| 2 (Brain)^[8]^ | Not measured | O (- 0.249 dB/cm) | X | X | X |
| 3 (Brain)^[7]^ | 73.7MPa | O (- 1.9 dB/cm) | X | X | X |
| 4 (Brain)^[12]^ | 45kPa | X | O (50kΩ) | X | X |
| 5 (Brain)^[22]^ | 4.8 & 39.4Mpa | O (- 0.94 dB/cm) | O (658kΩ) | X | X |
| 6 (PNS)^[52]^ | 30kPa | X | O (50kΩ) | X | X |

**Table S2.** Comparative summary of key material and functional properties of PUH-based thermally drawn all-hydrogel fibers versus representative hydrogel-based neural interfaces reported in the literature. Unlike prior systems that required separate assembly of optical, electrical, or fluidic components, the HG-TDP fibers integrate all three modalities within a single continuous architecture. References correspond to previously reported hydrogel-based interfaces.


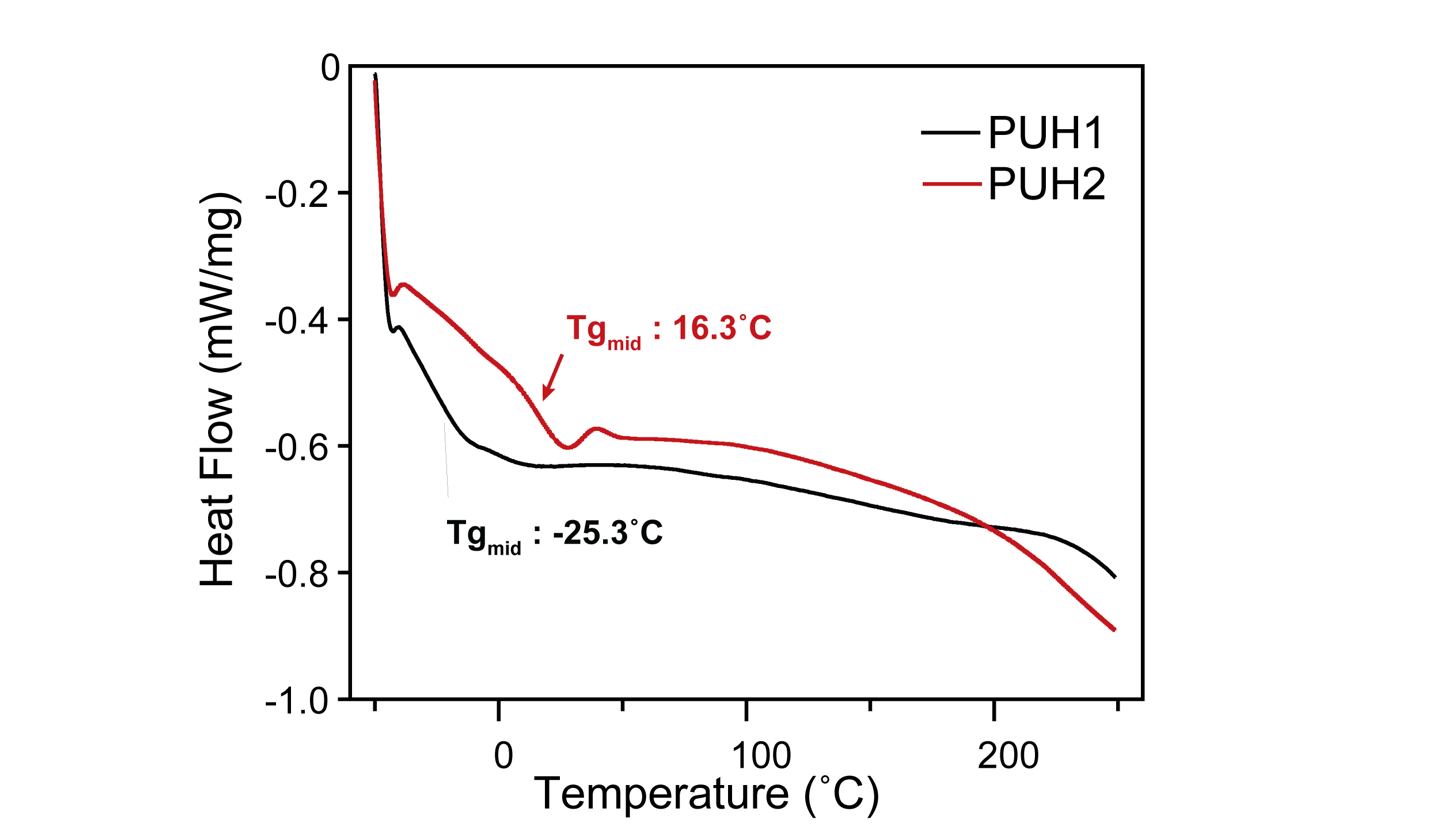


**Figure S1**. Differential scanning calorimetry thermogram of polyurethane hydrogels. Glass transition temperatures are marked on the graph. The specific heat capacities of PUH1 and PUH2 are 0.254 J/g·K and 0.130 J/g·K, respectively.


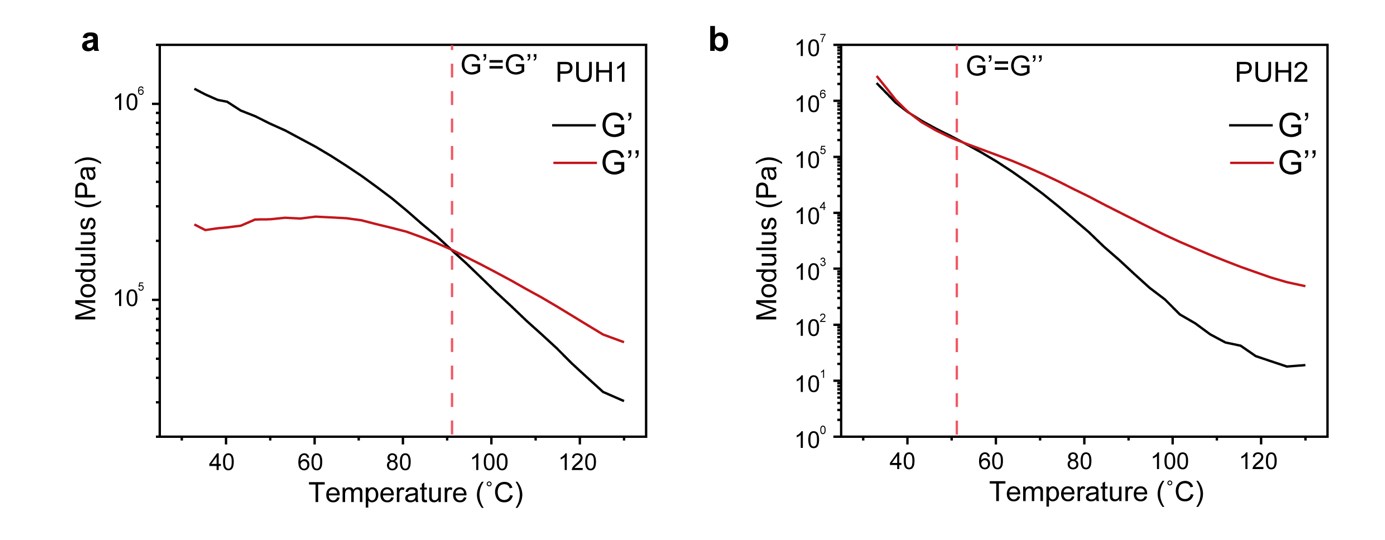


**Figure S2**. Rheology of thermoplastic hydrogels versus temperature. a–b) Storage (G’) and loss (G’’) modulus versus temperature for (a) PUH1 and (b) PUH2 hydrogels in their dry state. The red dashed line is used to indicate crossover point between the storage and loss modulus.


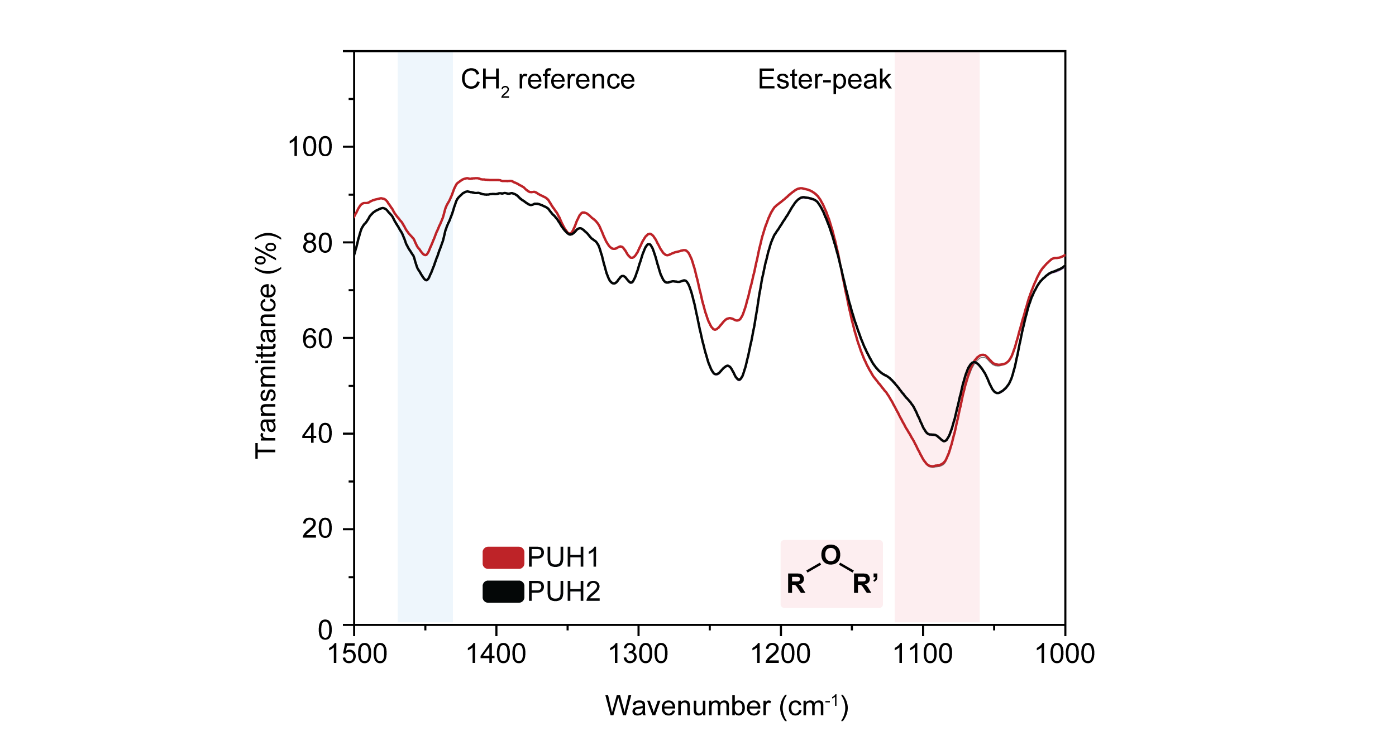


**Figure S3.** Fourier-transform infrared spectroscopy of thermoplastic hydrogels PUH1 and PUH2. Wavenumber ranges utilized in integration are shaded. Blue indicates the wavenumber range utilized for CH_2_ peak normalization (1470-1430 cm^-1^). Red indicates the wavenumber range utilized for the ether peak (1120-1060 cm^-1^).


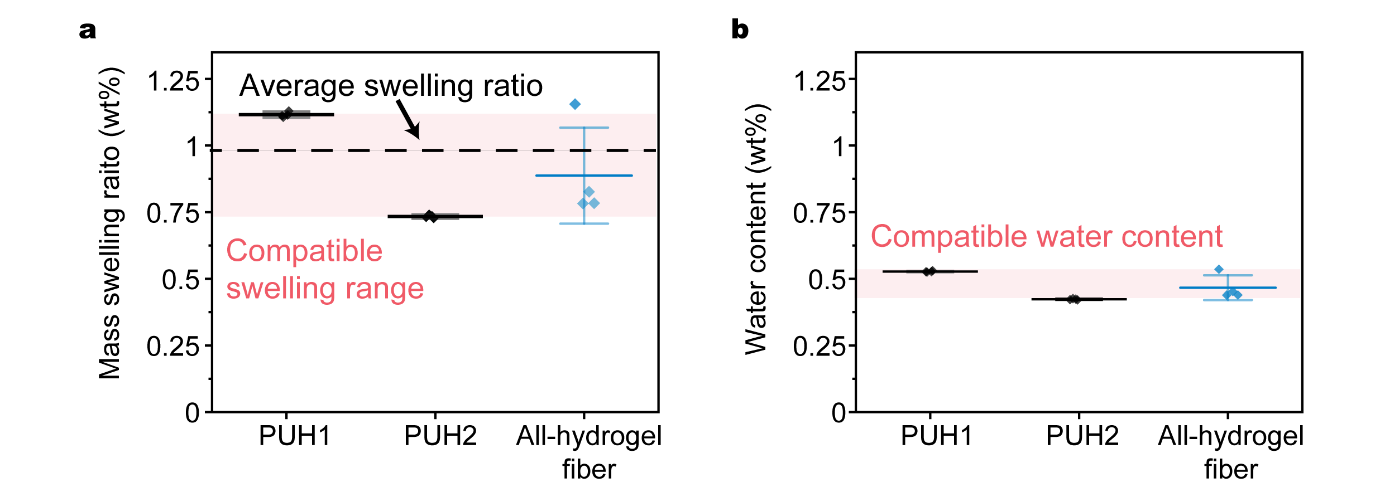


**Figure S4.** Swelling behavior of thermoplastic hydrogels (PUH1 and PUH2) and the all-hydrogel fiber. a) Mass water swelling ratio of thermoplastic hydrogels (PUH1, PUH2) and the all-hydrogel fiber. The mean swelling ratios are approximately 110 %, 70 %, and 90 % for PUH1, PUH2, and the all-hydrogel fiber, respectively. b) Mass water content of thermoplastic hydrogels (PUH1, PUH2) and all-hydrogel fiber. The mean water contents are approximately 52%, 42 %, and 47% for PUH1, PUH2, and the all-hydrogel fiber, respectively Values in a–b represent the mean and standard deviation (*n* = 4 for each all groups).


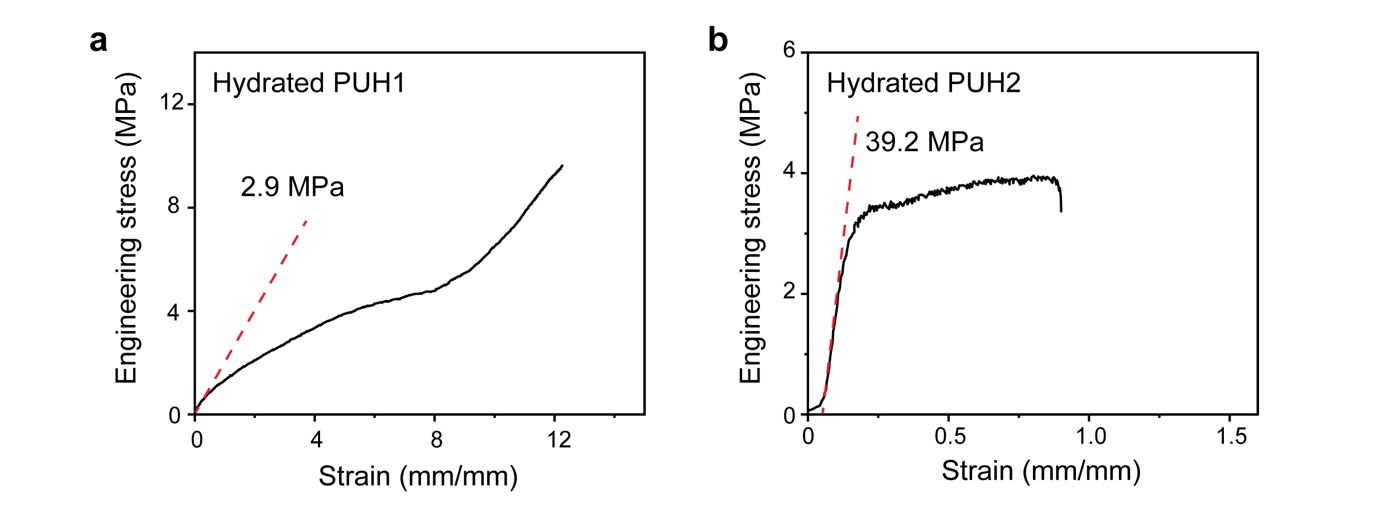


**Figure S5**. Tensile Young’s modulus of thermoplastic hydrogels. a) Engineering stress vs strain of PUH1 after hydration (*n* = 6, representative curve is shown). The tangent line used to measure Young’s modulus is represented as a red dashed line. b) Engineering stress vs strain of PUH2 after hydration (*n* = 6, representative curve is shown).


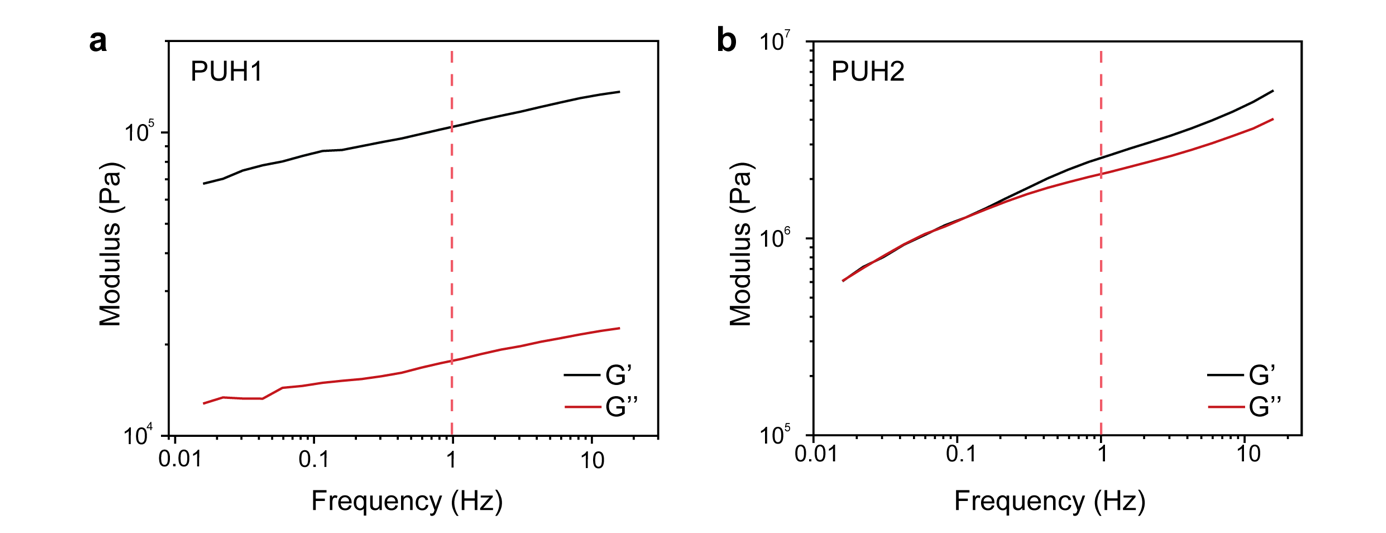


**Figure S6**. Shear modulus of thermoplastic hydrogels. a–b) Storage (G’) and loss (G’’) modulus versus frequency for (a) PUH1 and (b) PUH2 after hydration. The red dashed line is used to indicate the shear modulus at 1 Hz.


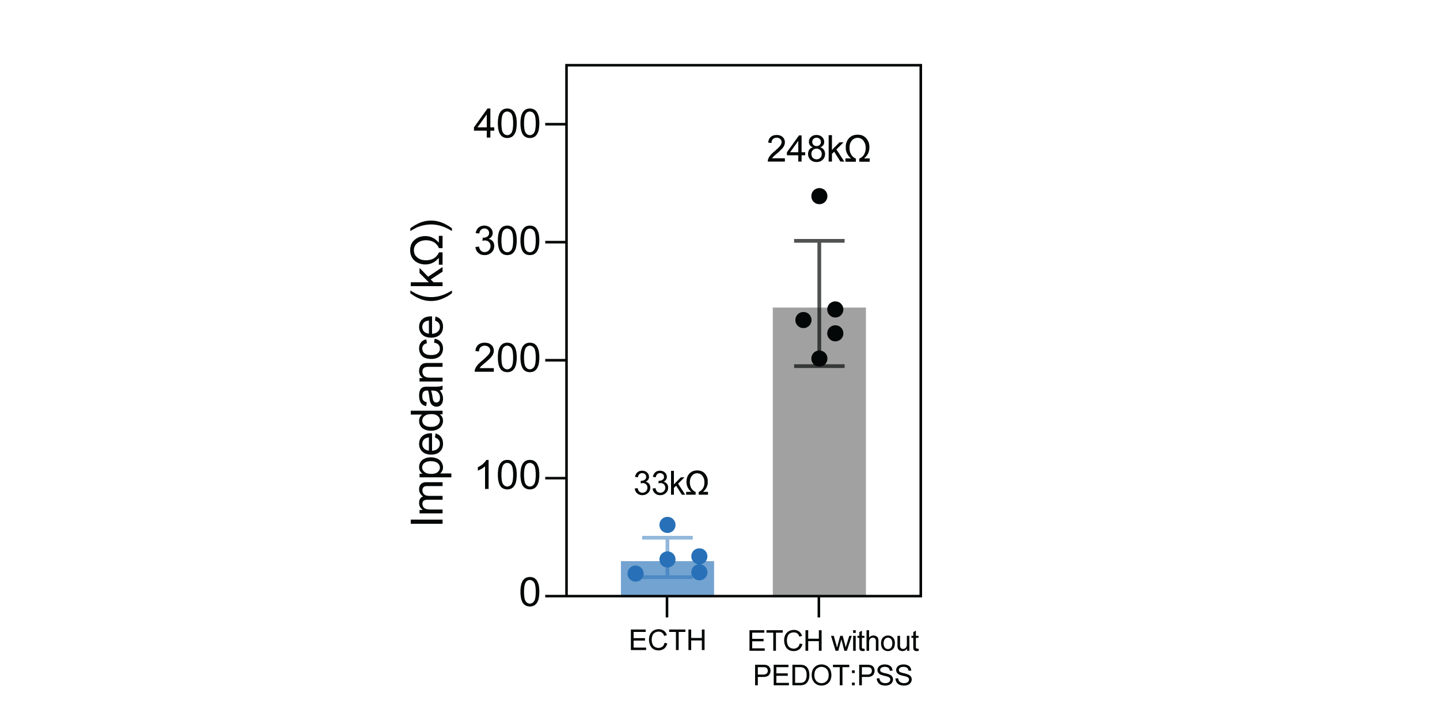


**Figure S7.** Electrical conductivity of the electrically conductive thermoplastic hydrogel (ECTH) utilized in the electrode of the all-hydrogel fiber. Impedance at 1 kHz of the ECTH (PUH2/rGO 4 wt%/PEDOT:PSS 0.5 wt% composite) and the ECTH without PEDOT:PSS (PUH2/rGO 4 wt% composite).


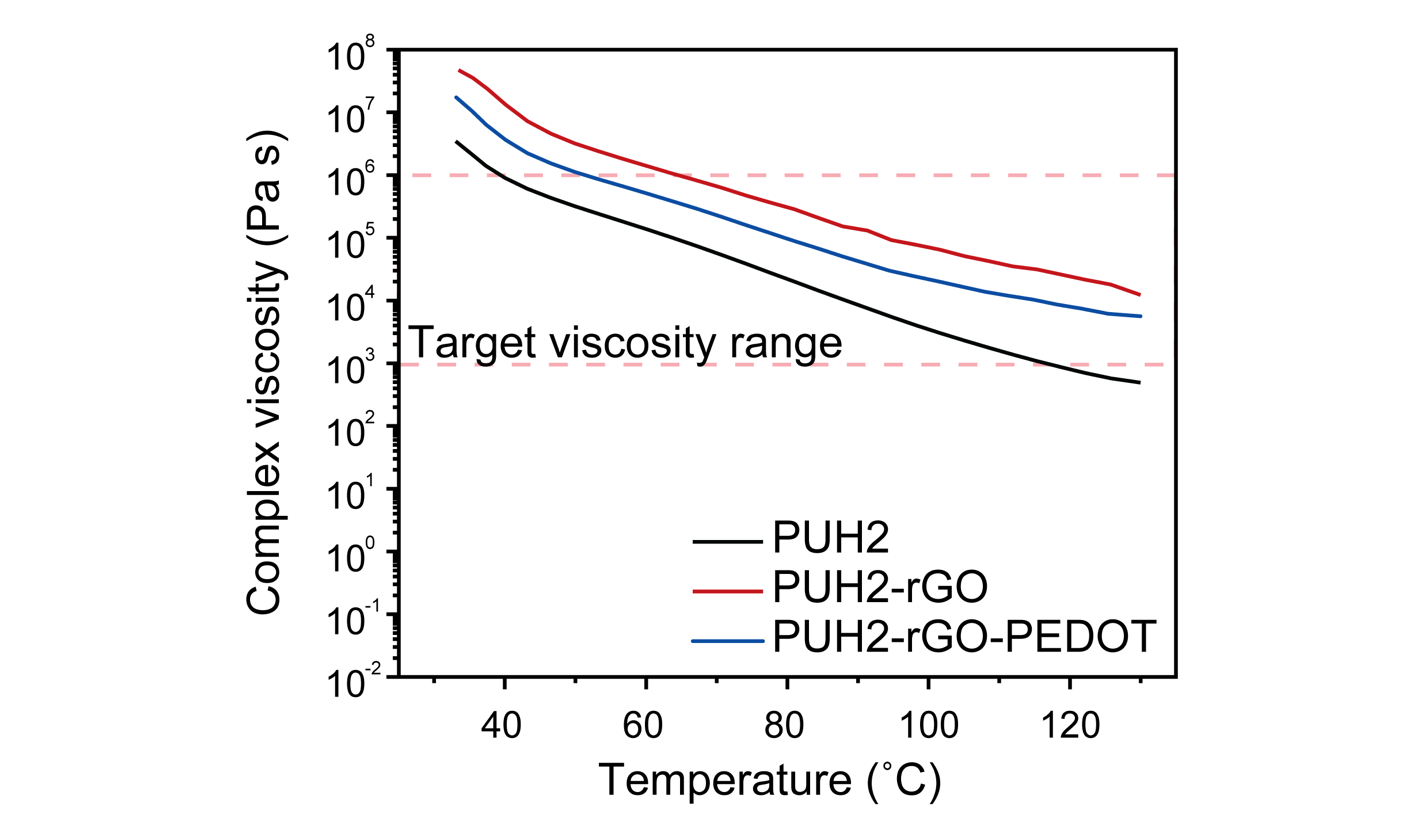


**Figure S8**. Rheology of thermoplastic electrode hydrogels with temperature. Storage (G’) and loss (G’’) modulus versus temperature for PUH2, PUH2-reduced graphene oxide (rGO) composites, and PUH2-rGO-PEDOT:PSS composites in their dry state. The red dashed line indicates the viscosity range compatible with the thermal drawing process.


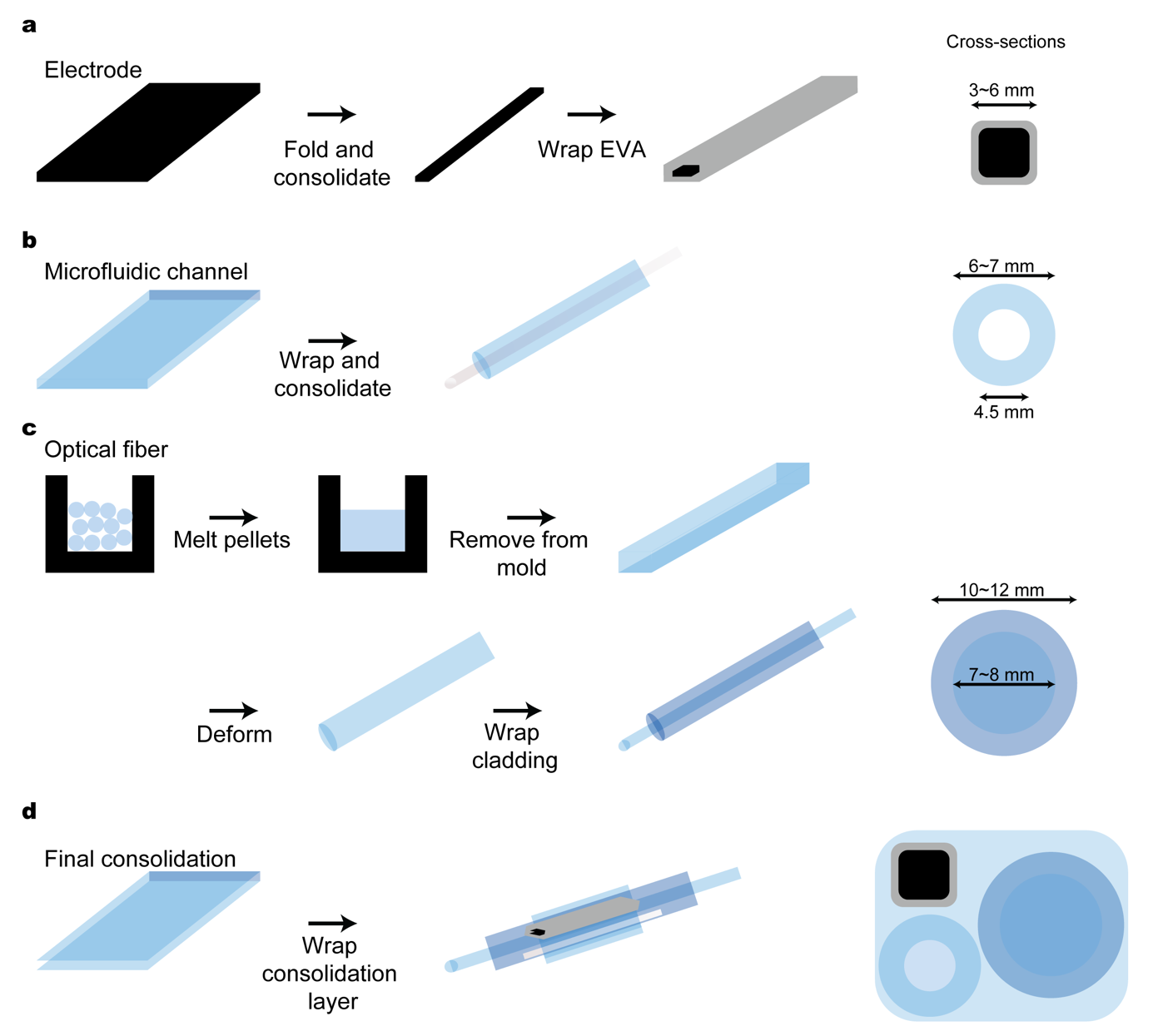


**Figure S9.** Preform fabrication protocols for each fiber type integrated into HG-TDP. a**–**c) Schematics of the preform preparation methods of (a) individual electrode, (b) microfluidic channels and (c) optical fiber preforms prepared with thermoplastic hydrogels (PUH1 and PUH2), the ETCH, and thermoplastic insulator, EVA. d) Schematic of final consolidation process. The individual preforms are consolidated into an integrated multifunctional all-hydrogel preform before the thermal drawing process.


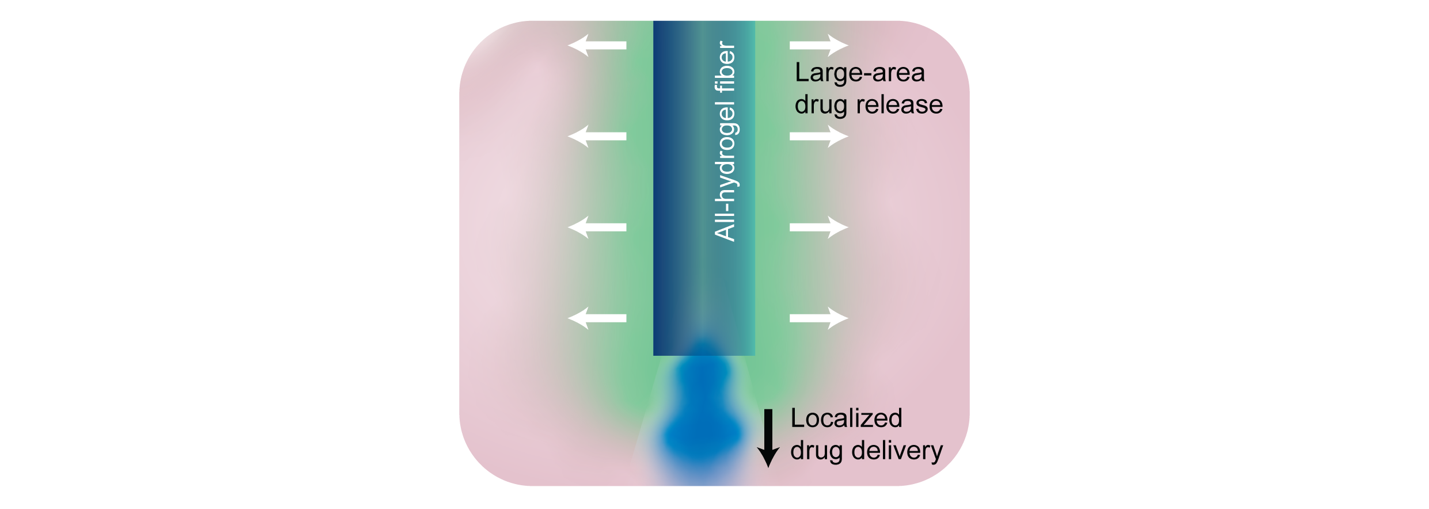


**Figure S10.** Drug delivery mechanisms in the all-hydrogel fibers. The all-hydrogel fibers demonstrate two independent drug delivery mechanisms: localized drug delivery and pre-implantation loaded large-area drug release, with distinct coverage regions during chemical interfacing and release mechanisms. Localized drug delivery is based on convection enhanced diffusion from the tip of the fiber.^[53]^ Pre-implantation loaded large area drug release is based on small molecule drugs embedded in the hydrogel matrix and released from the longitudinal side of the fiber.^[54]^


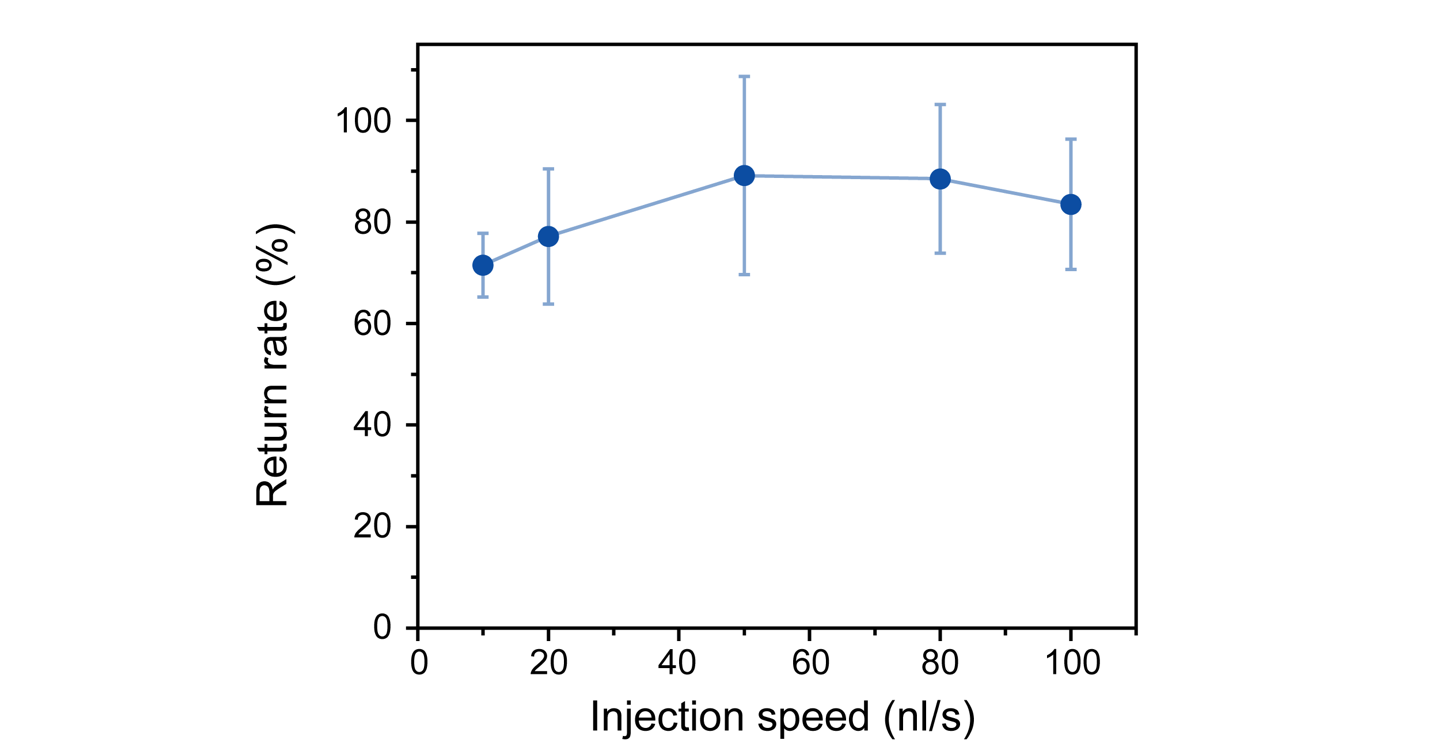


**Figure S11**. Evaluation of microfluidic channel performance. Return rate of all-hydrogel fibers at various injection speeds. Values represent the mean and standard deviation (*n* = 5).


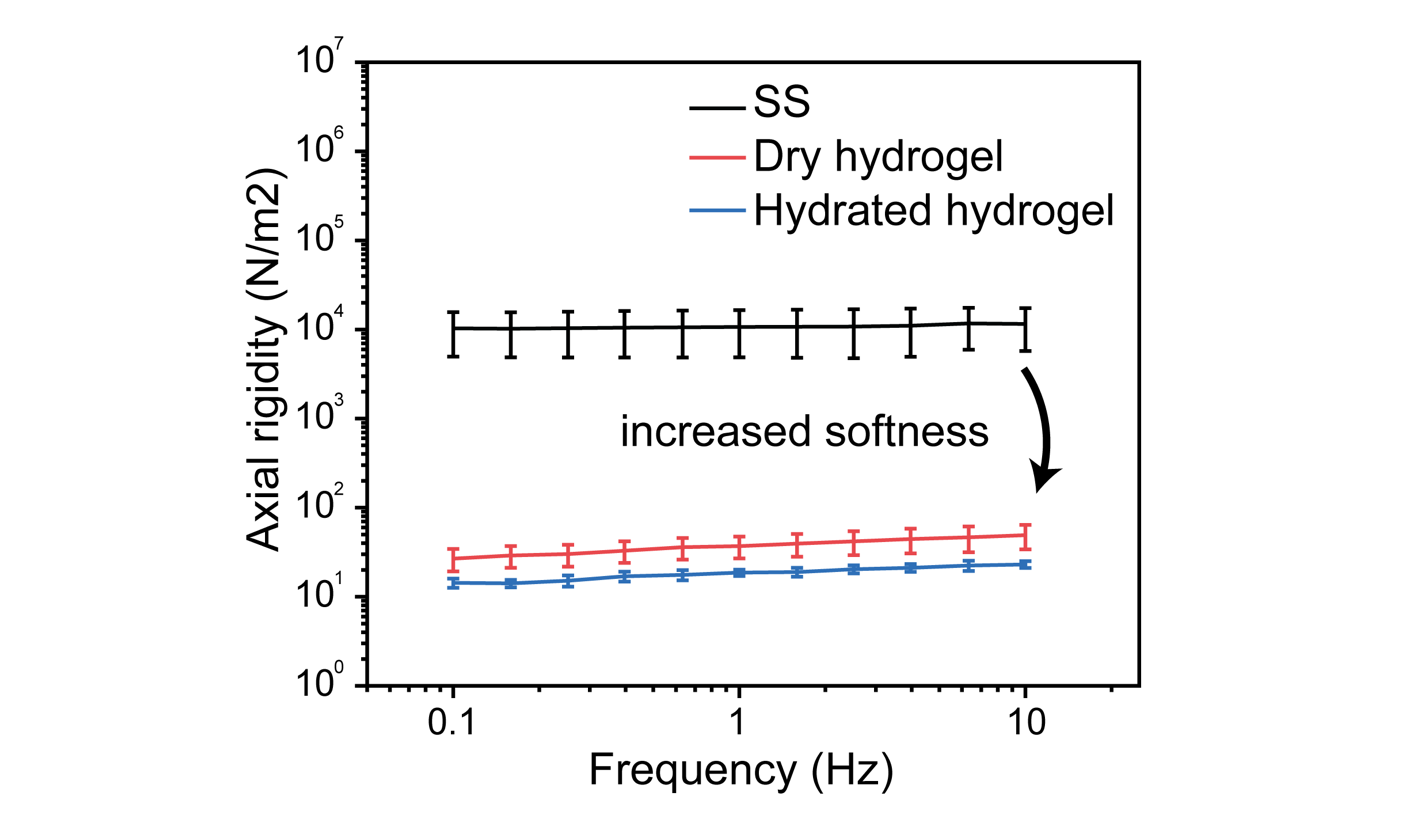


**Figure S12**. Axial rigidity of dry and hydrated all-hydrogel fibers in comparison with stainless-steel fibers (400 µm outer diameter) at various frequencies. Values represent the mean and standard deviation (*n* = 3 for stainless steel, *n* = 6 for hydrogel fibers)


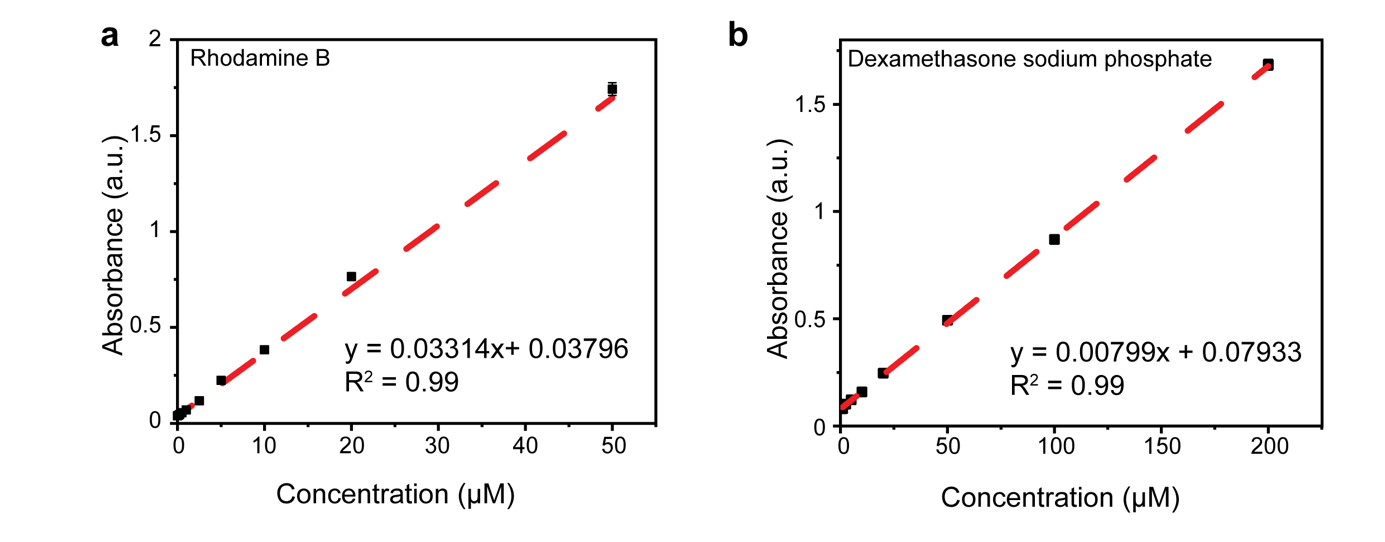


**Figure S13**. Calibration curves for large-area chemical interfacing from all-hydrogel fibers. (a) Rhodamine B and (b) dexamethasone sodium phosphate for quantification of drug concentration, as measured with a UV–Vis spectrometer. Values represent the mean and standard deviation (*n* = 4). The calibration equation and R^2^ values are shown on the graphs.


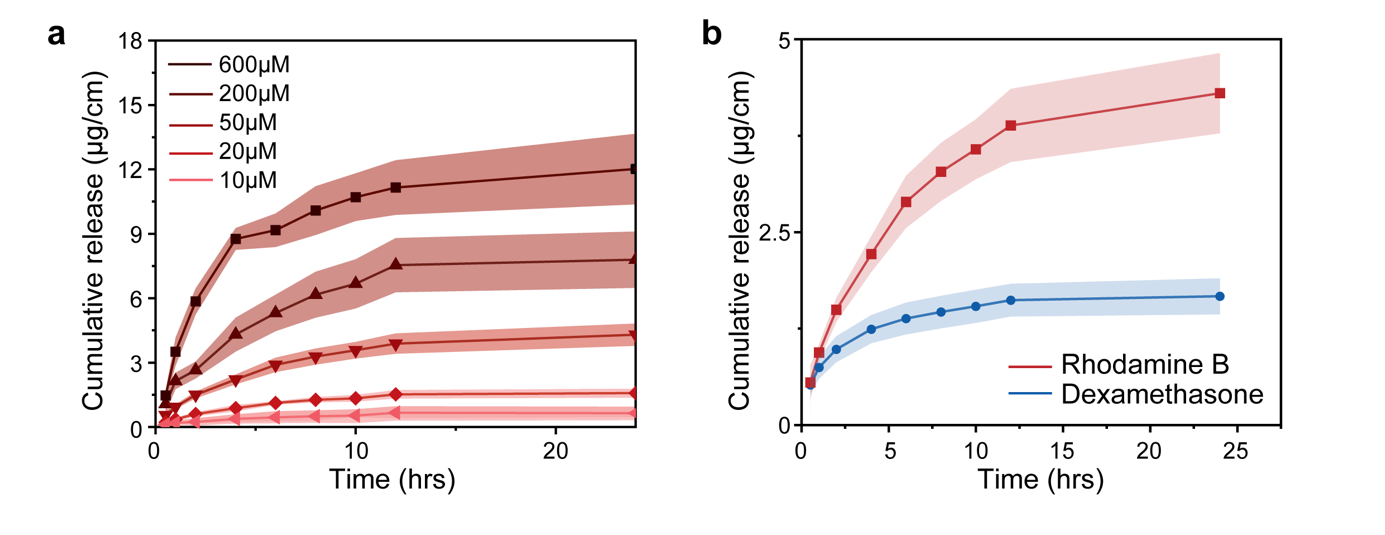


**Figure S14**. Large-area drug release of all-hydrogel fibers. a) Cumulative release curves for all-hydrogel fibers after incubation in various concentrations of rhodamine B loading baths. b) Cumulative release curves over 24 hours of rhodamine B and dexamethasone sodium phosphate from all-hydrogel fibers. Values represent the mean and standard deviation (*n* = 5­ – 6).


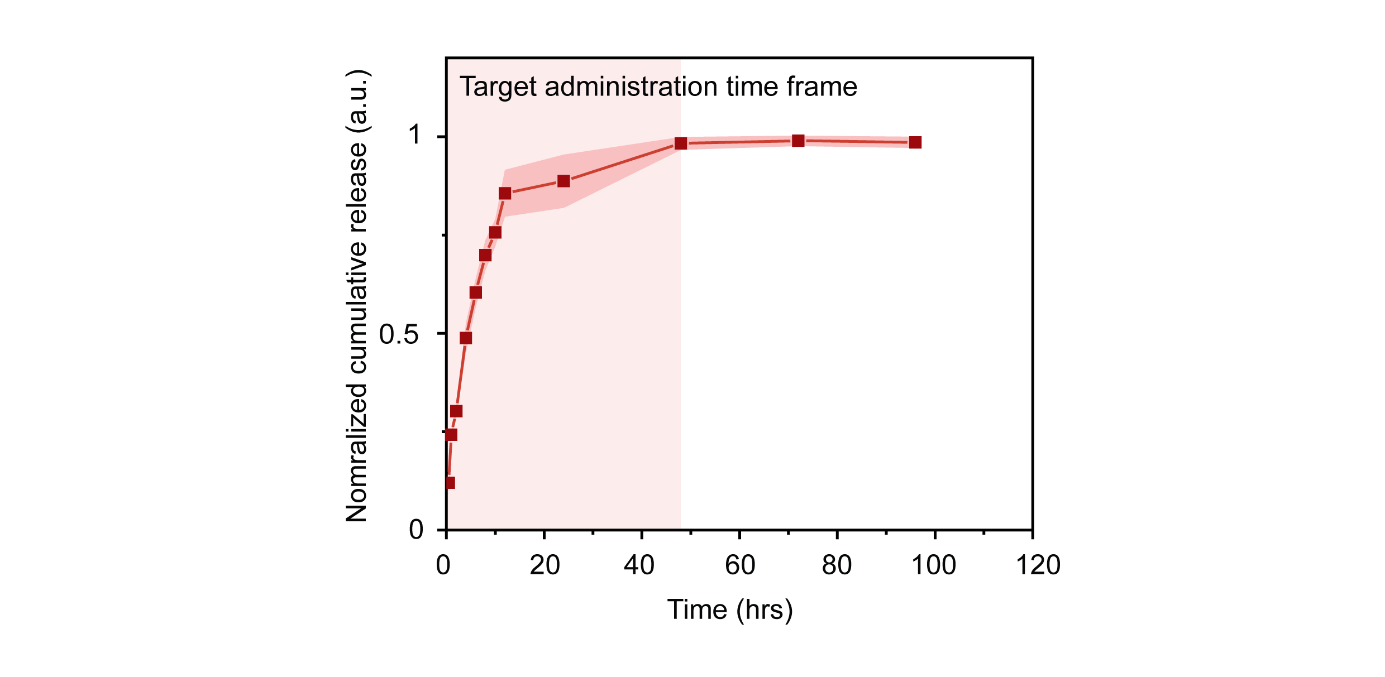


**Figure S15.** Large area drug release of the all-hydrogel fiber over 96 hours. Normalized cumulative release profile of a small molecule drug (Rhodamine B) across 4 days (*n* = 6). The target administration time frame of dexamethasone is shaded in red. Data points and shaded area represent mean and standard deviation respectively.


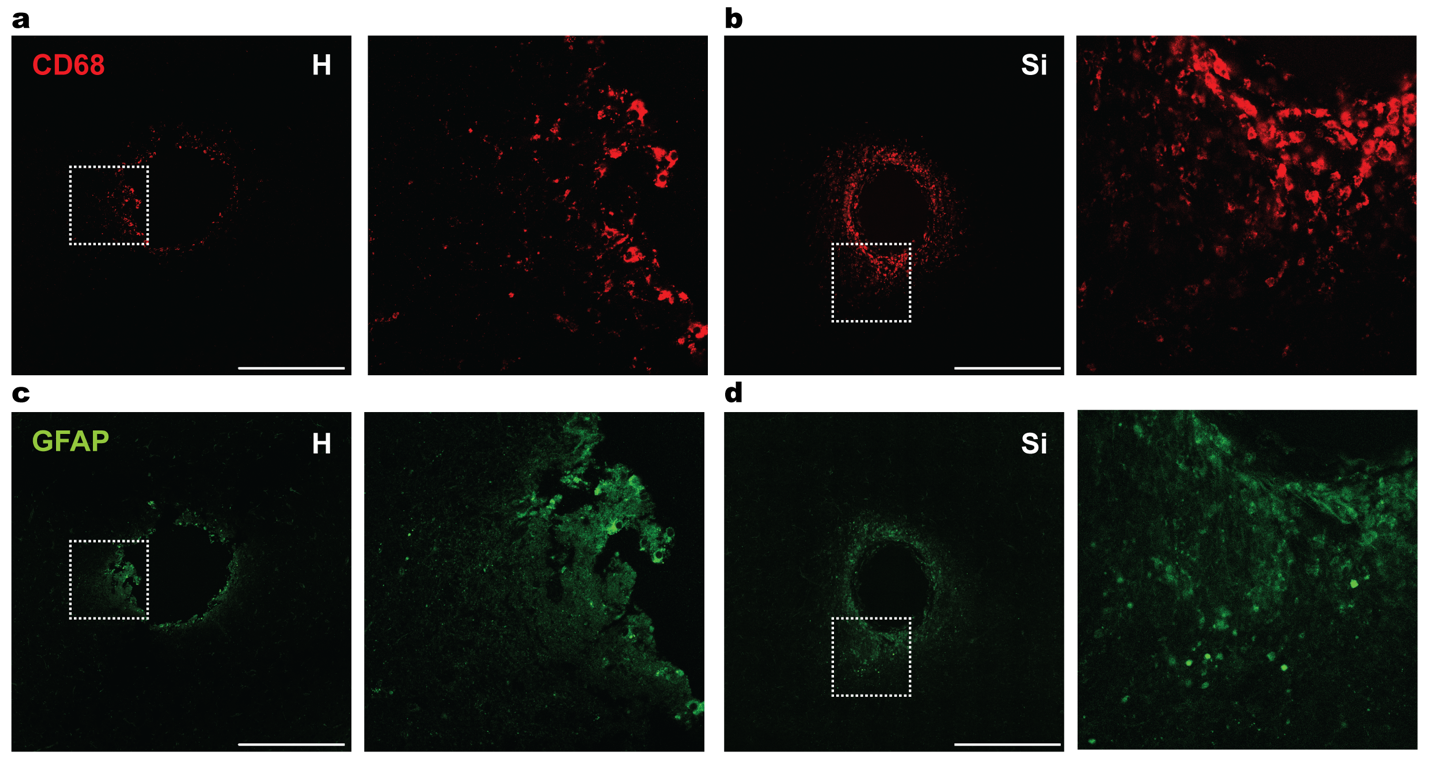


**Figure S16**. Immune response images of all-hydrogel fibers (H) in comparison with commercial neural implants. Silica (Si) optical fibers are utilized as commercial neural implants. a–b) Representative confocal image (left) and high magnification inset (right) of activated macrophages (CD68) surrounding the (a) all-hydrogel fiber and (b) silica fiber. c–d) Representative confocal image (left) and high magnification inset (right) of astrocytes (GFAP) surrounding the (c) all-hydrogel fiber and (d) silica fiber. Scale bars represent 400 µm.


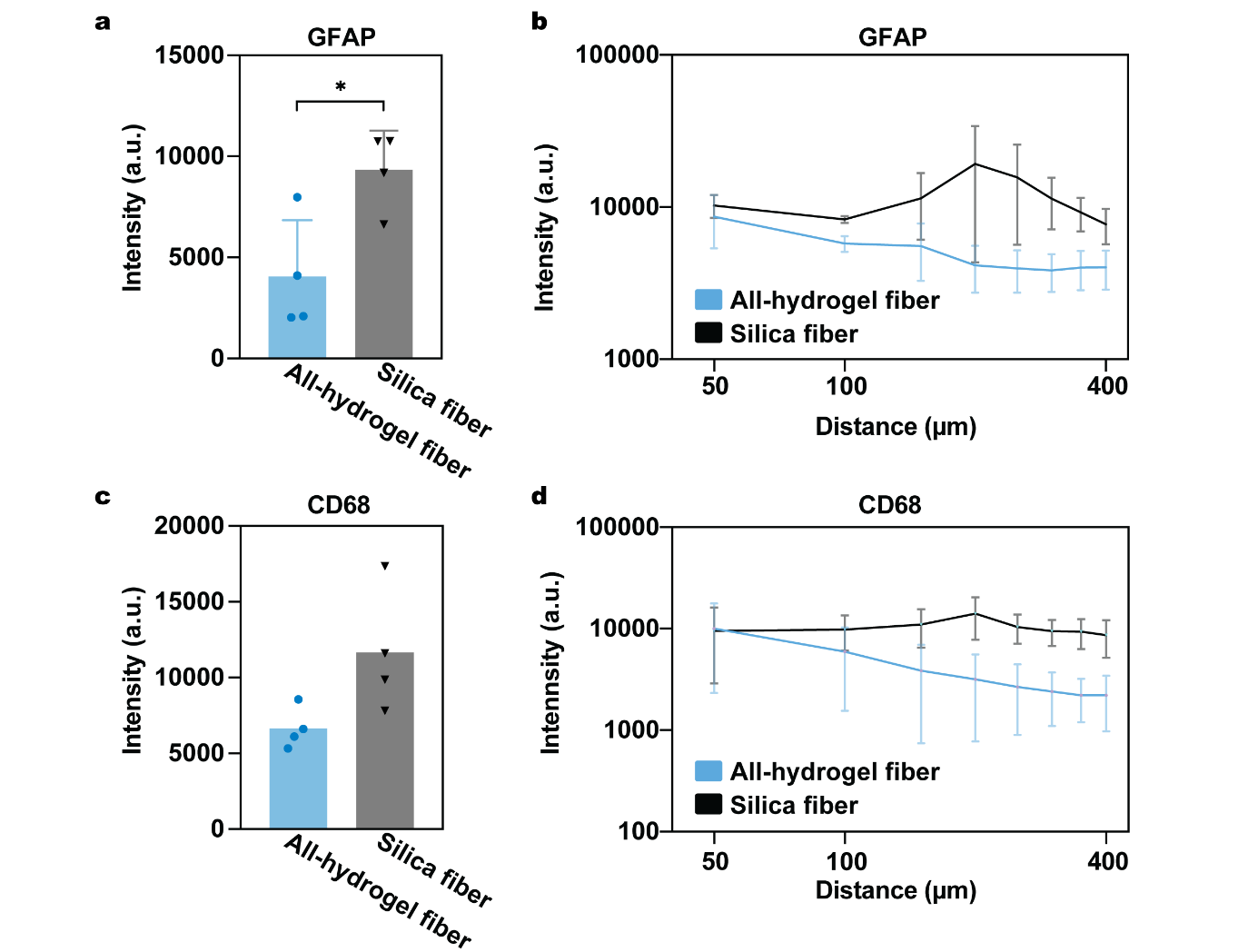


**Figure S17.** Immunohistochemistry of all-hydrogel fibers in comparison with optical fibers utilized for neural implants. (a–b) Comparison of astrocyte (GFAP) (a) intensity (*P* = 0.021) and (b) intensity distance profiles in all-hydrogel fibers and silica fibers. (c–d) Comparison of activated macrophage (CD68) (c) intensity (*P* = 0.060) and (d) intensity distance profiles in all-hydrogel fibers and silica fibers. Values represent the mean and standard deviation (number of animals, *n* = 4, **P* < 0.05; Two-sided t-test).

**
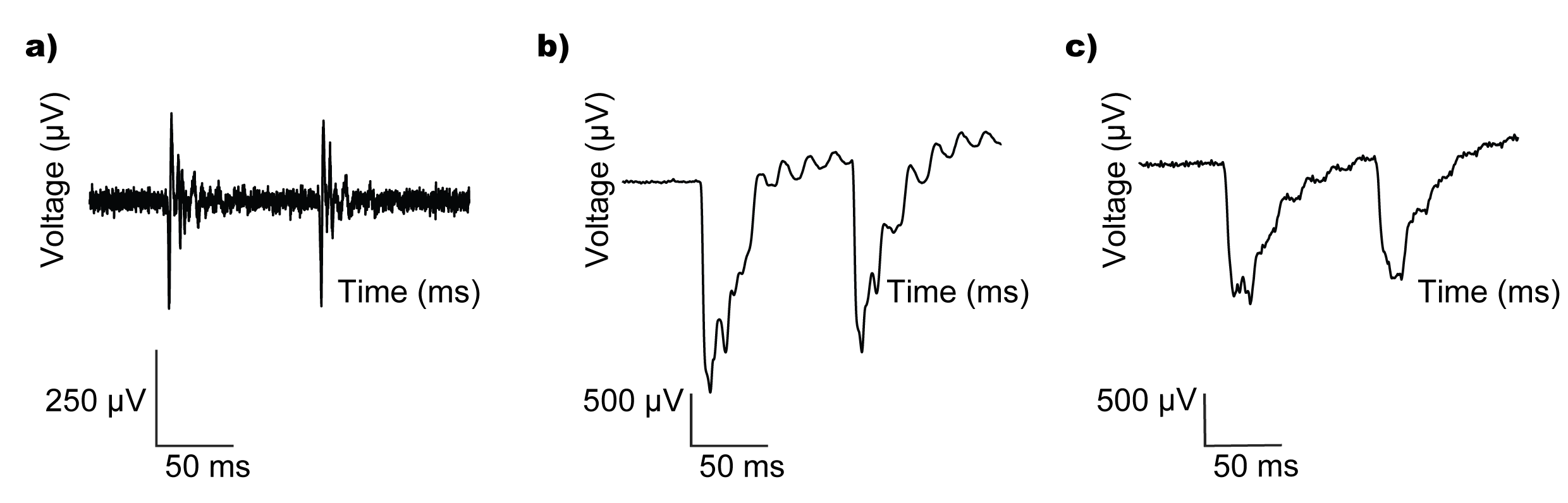
**

**Figure S18.** Representative waveforms of optically-evoked potentials. a–b) Representative waveforms of (a) multi-unit and (b) local field potentials recorded with the insulated all-hydrogel fiber. c) Representative waveforms of local field potentials recorded with the uninsulated all-hydrogel fiber.


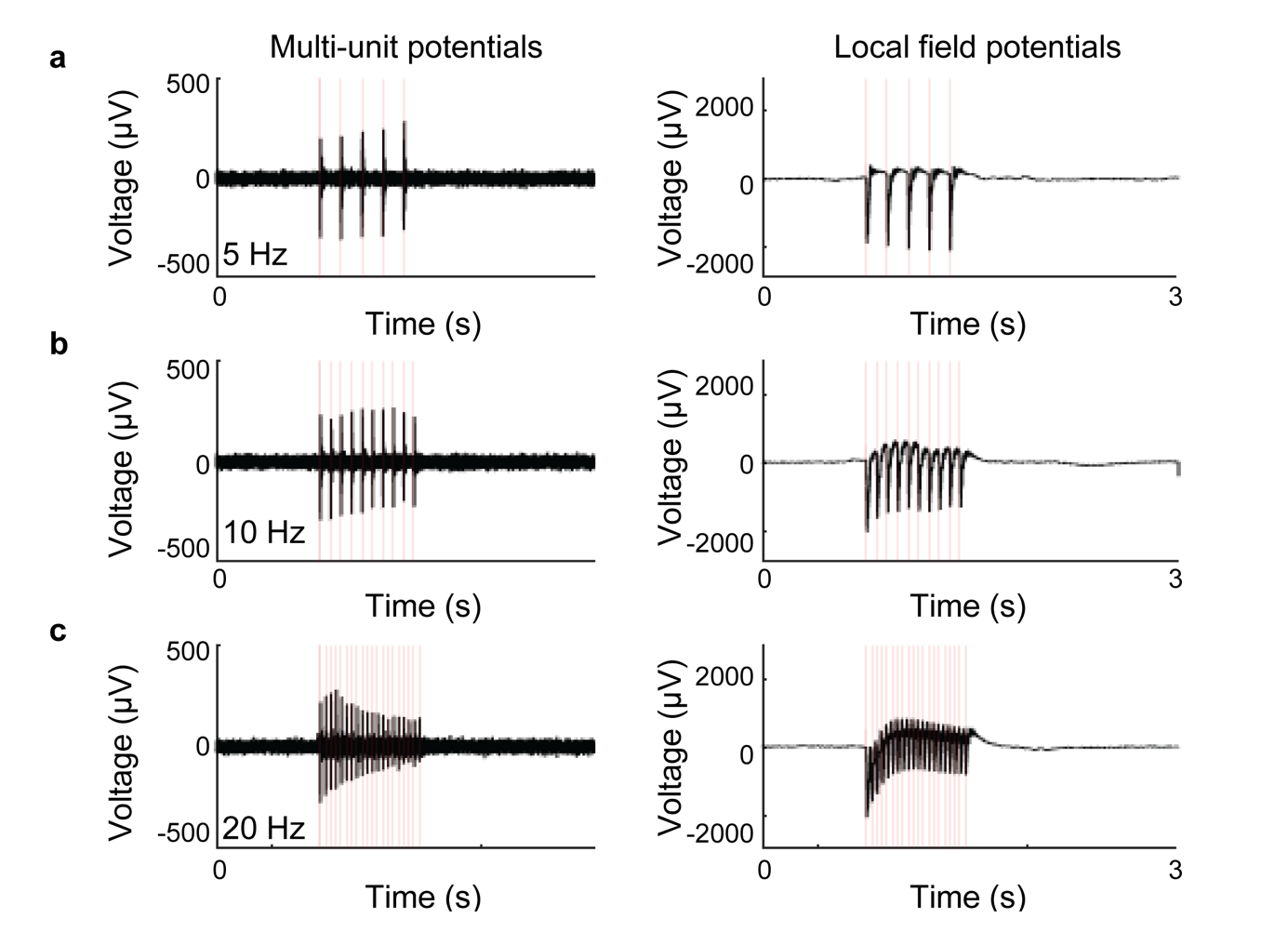


**Figure S19**. Optically-evoked potentials with all-hydrogel fibers at stimulation frequencies of (a) 5 Hz, (b) 10 Hz and (c) 20 Hz. Multi-unit potentials are plotted on the left and local field potentials are plotted on the right of each subfigure. Red lines indicate pulses of optical stimulation.


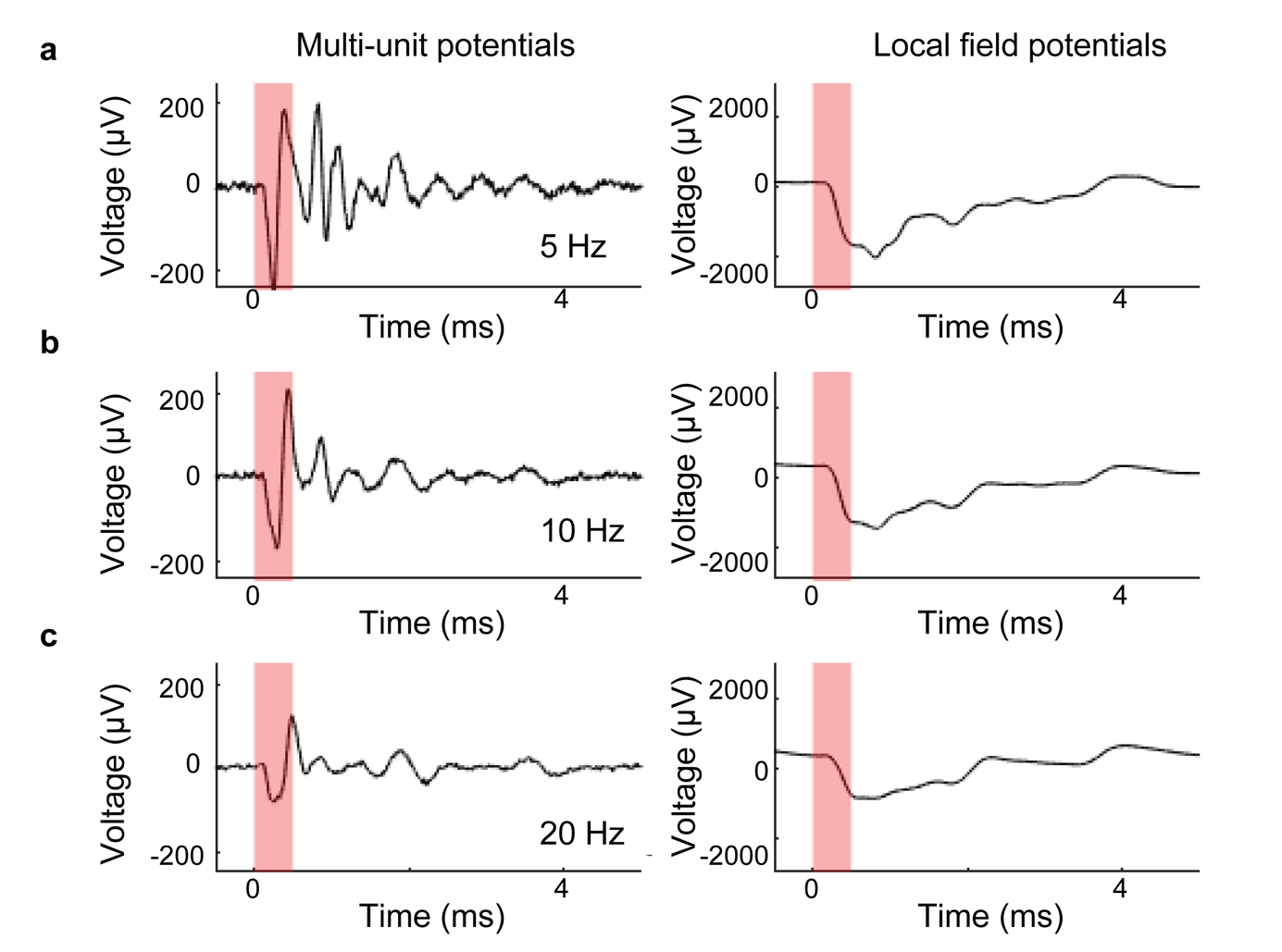


**Figure S20**. Average waveform of optically-evoked potentials. Waveforms demonstrate neural activity after the optical stimulus finishes. Optically-evoked potentials at (a) 5 Hz, (b) 10 Hz and (c) 20 Hz are recorded. Multi-unit potentials are plotted on the left and local field potentials are plotted on the right of each subfigure. Red area indicates the onset of optical stimulation.


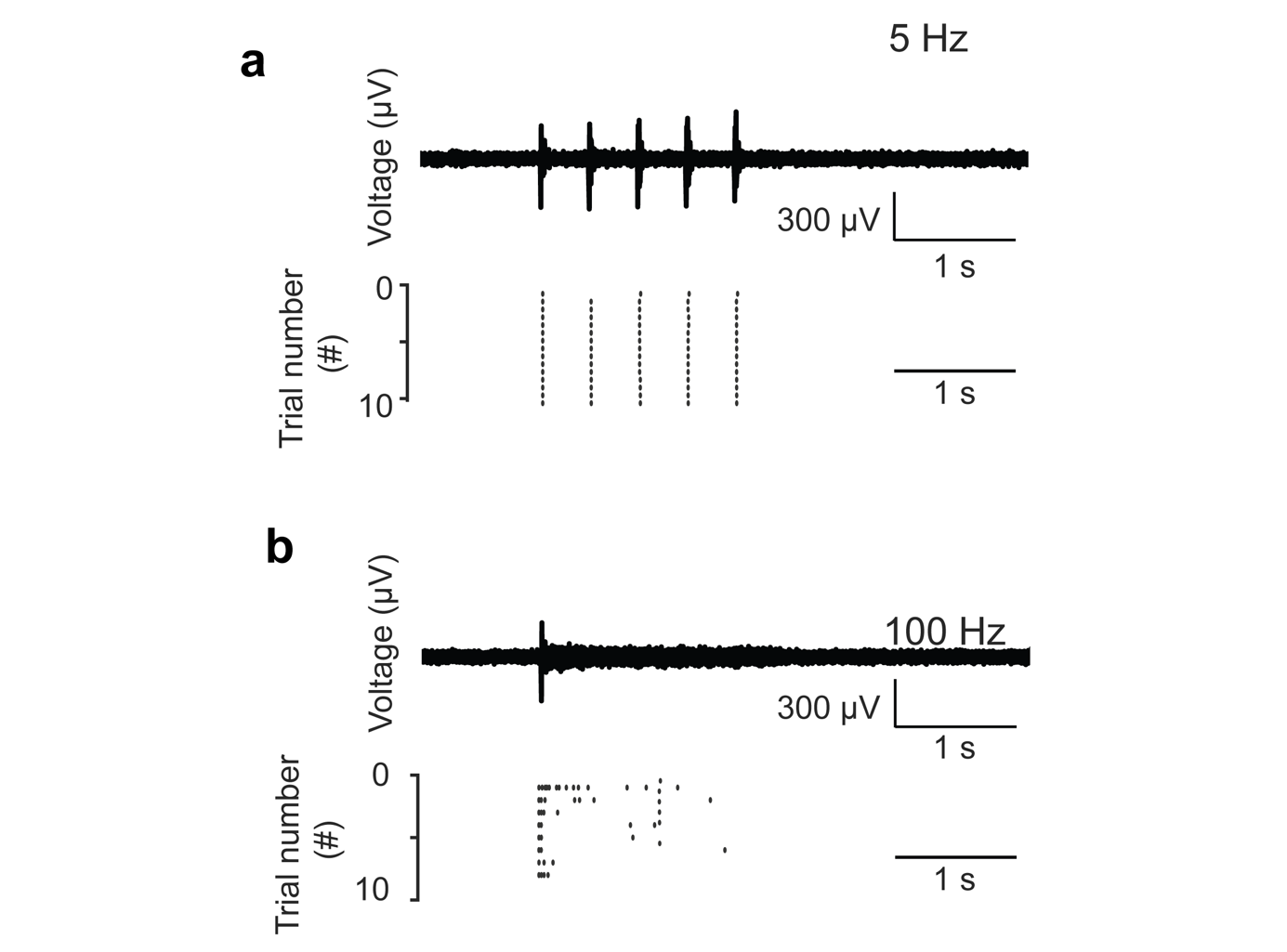


**Figure S21**. Raster plot of optically-evoked multi-unit activity with all-hydrogel fibers across 10 stimulation trials at (a) 5 Hz and (b) 100 Hz. Stimulation was successful at all pulses at 5 Hz, while Channelrhodopsin-2 failed to follow 100 Hz stimulation.


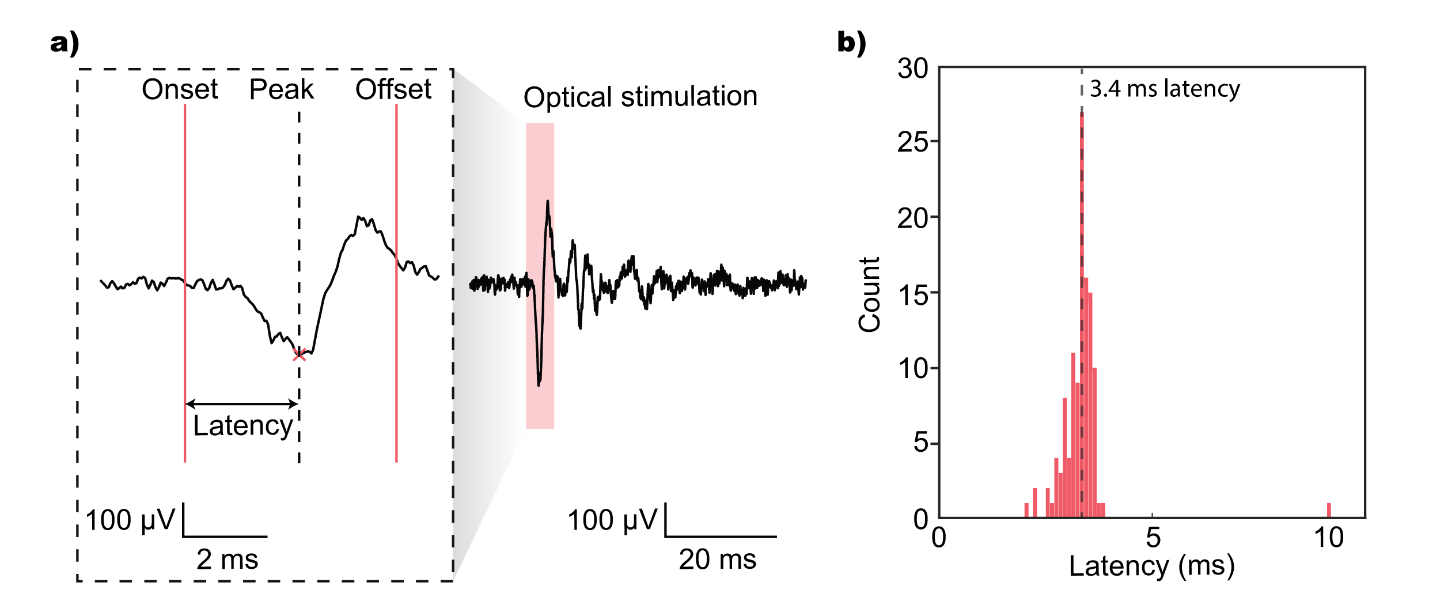


**Figure S22**. Optically-evoked multi-unit potentials of insulated all-hydrogel fibers. a) Representative waveform of optically-evoked multi-unit potentials recorded with the insulated all-hydrogel fiber. (left) Optically-evoked potential during the stimulation pulse. The onset and offset times of the optical stimulation pulse is marked with red vertical lines. (right) Optically-evoked potential during and after the stimulation pulse. The duration of the optical stimulation pulse is marked with the shaded square. b) Histogram of the latency of the optically-evoked potentials. Latency is calculated by measuring the time difference between the onset of optical stimulation and the recorded potential peak of multi-unit activity.


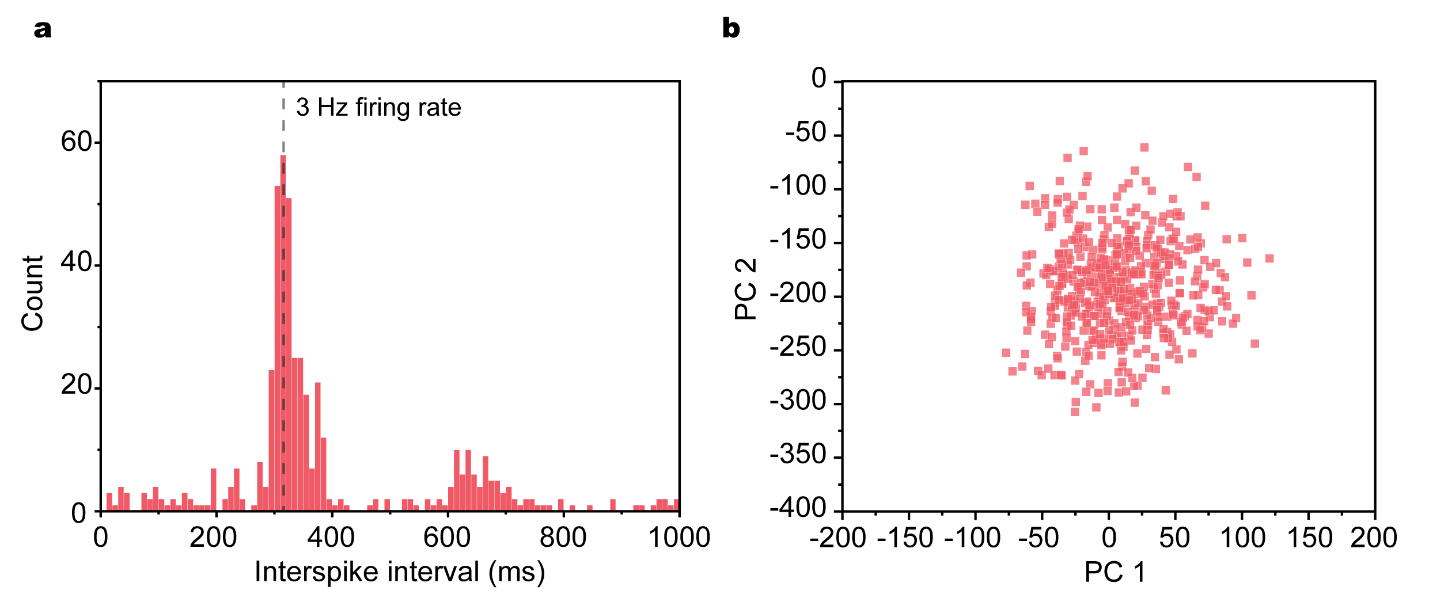


**Figure S23.** Single-unit spiking data in positive amplitude spikes. a) Interspike-interval (ISI) histogram of the positive amplitude spikes from 517 detected spikes. Bin width is 10 ms and the sorted neuron demonstrates a 3 Hz spiking rate. b) Principal component analysis clustering of the positive amplitude spikes suggests a single cluster.


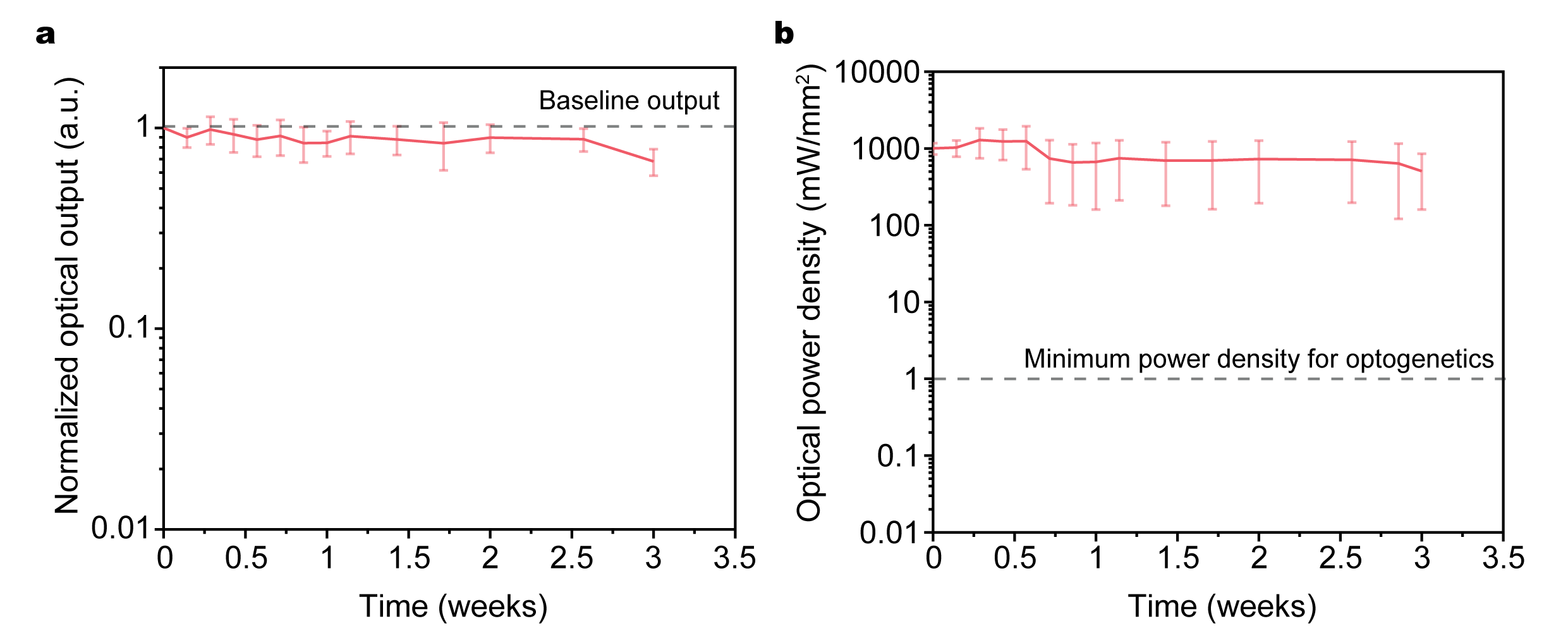


**Figure S24.** In vitro stability of the hydrogel optical waveguide over 3 weeks. a) Normalized optical output versus time. The dashed line indicates the baseline optical output. b) Optical power density versus time is measured. The dashed line indicates the minimum power density required for optogenetic stimulation 1 mWmm^-2^.^[55]^ Fiber samples are incubated at 37˚C in a saline solution to mimic environments with hydrolytic degradation in vivo. Values represent mean and standard deviation (*n* = 4 for each time point).


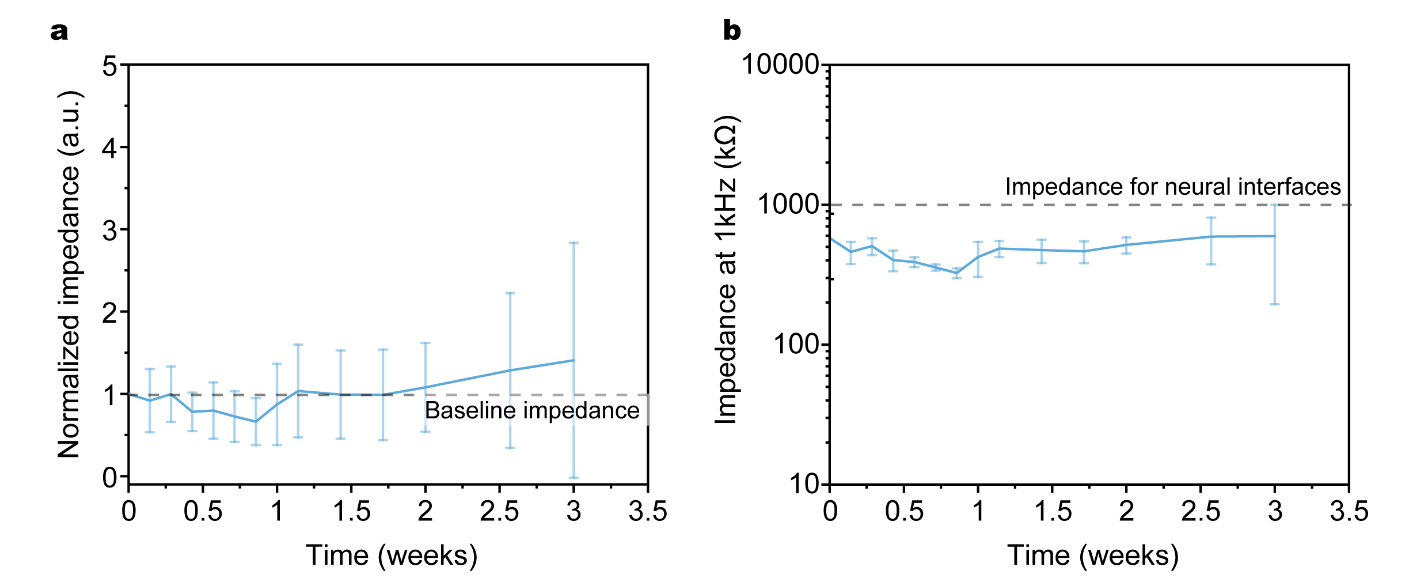


**Figure S25.** In vitro stability of the hydrogel electrode over 3 weeks. a) Impedance at 1kHz versus time. The dashed line indicates the baseline electrical impedance. b) Normalized impedance at 1 kHz versus time. The dashed line indicates the impedance requirement for implantable neural interfaces.^[56]^ Fiber samples are incubated at 37˚C in a saline solution to mimic environments with hydrolytic degradation in vivo. Values represent mean and standard deviation (*n* = 4 for each time point).


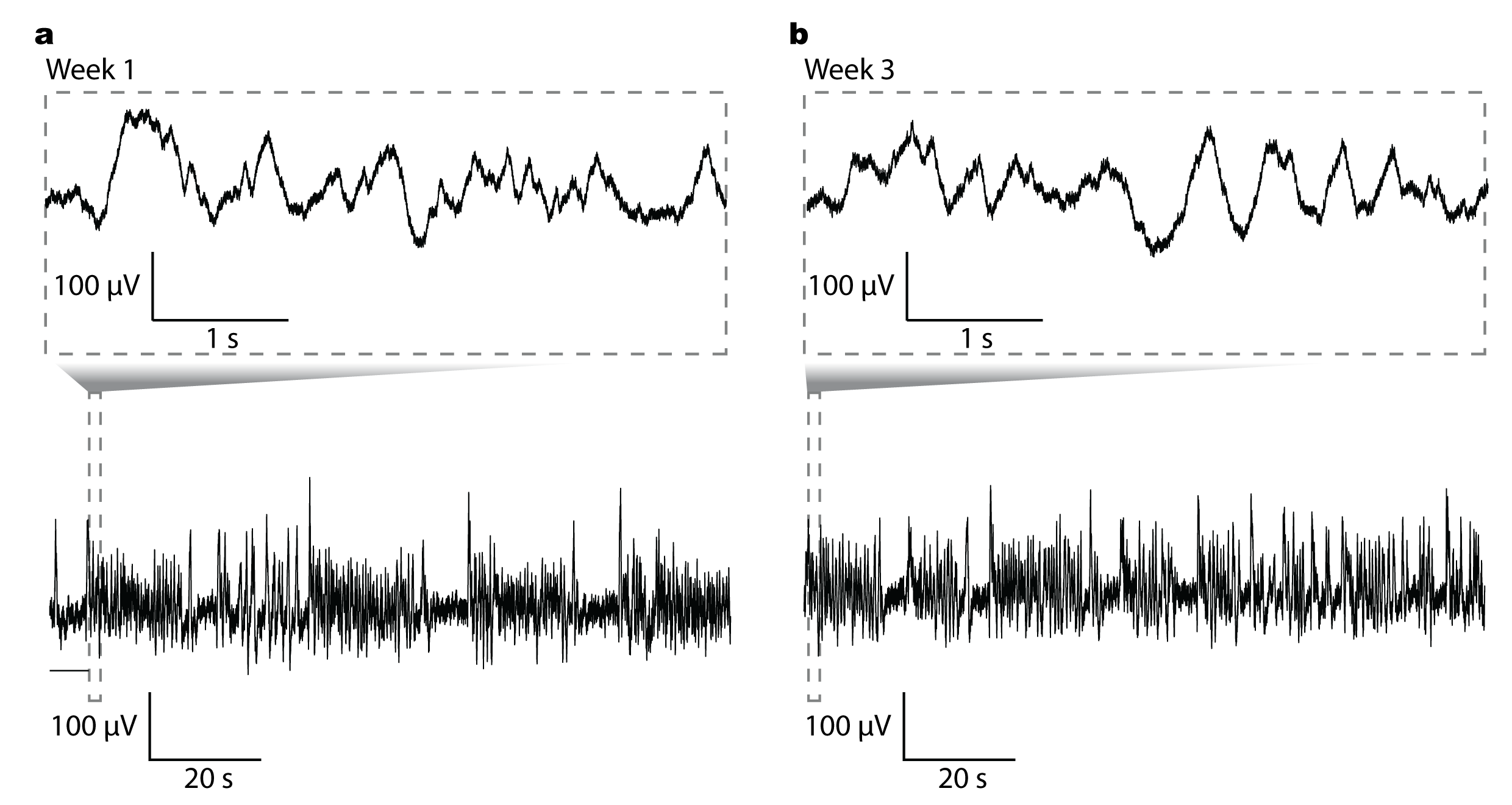


**Figure S26.** Long-term endogenous electrophysiology with all-hydrogel fibers. a–b) Electrophysiological signals at (a) 1 week and (b) 3 weeks in lightly anesthetized mice (0.75 % isoflurane). Bottom traces show stable recording over five minutes. Top insets show 5 second representative waveforms.


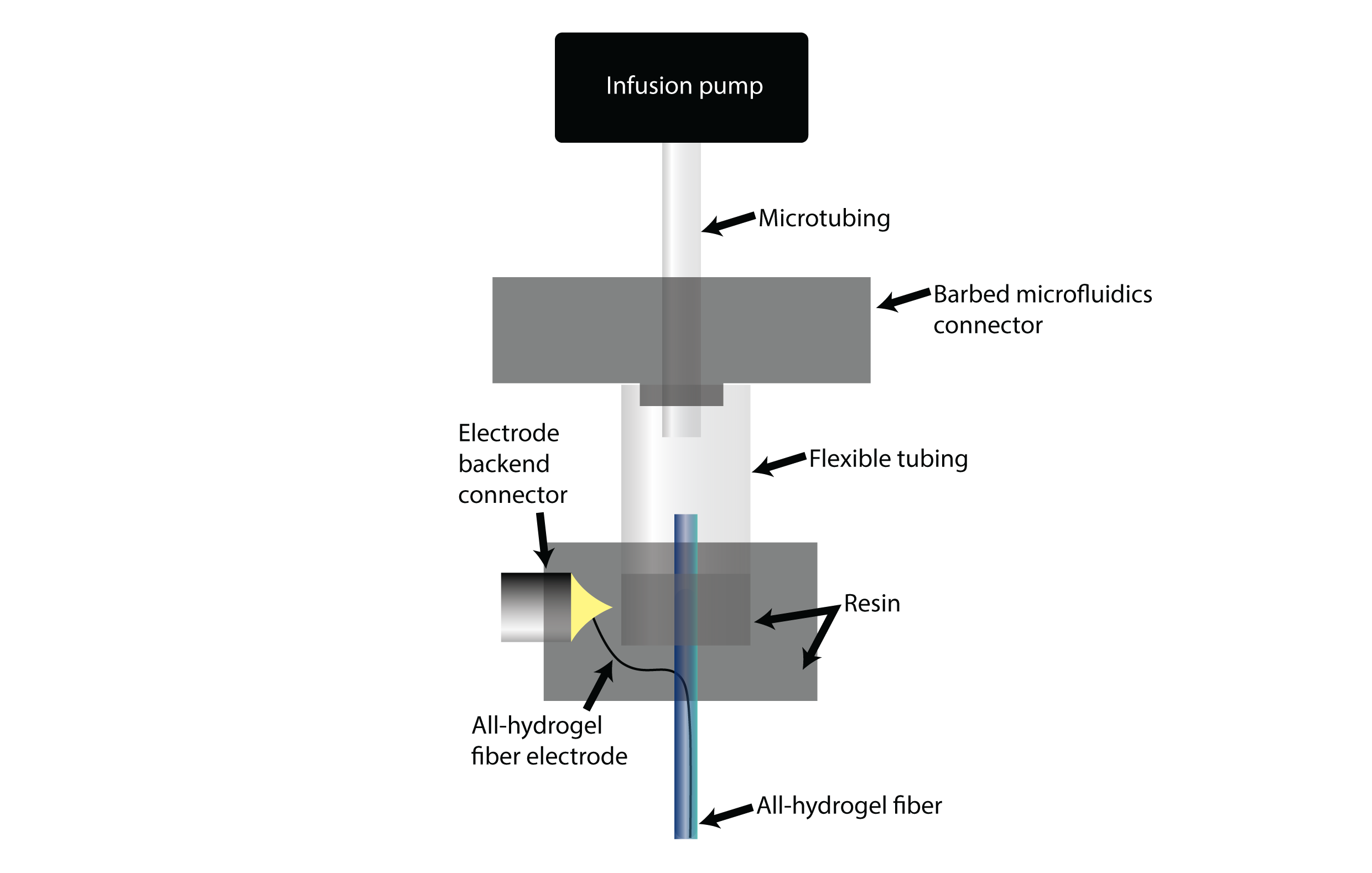
 **Figure S27.** Backend connection of the microfluidic channels to the infusion pump with multifunctional all-hydrogel fibers.
